# Supplementary material for: The Ustilago maydis repetitive effector Rsp3 blocks the antifungal activity of mannose-binding maize proteins
Source: Nat Commun. 2018 Apr 27;9:1711. doi: 10.1038/s41467-018-04149-0 (PMC5923269; doi:10.1038/s41467-018-04149-0)
Supplement: Supplementary file 1 — Supplementary Information [file 41467_2018_4149_MOESM1_ESM.pdf]

The *Ustilago maydis* repetitive effector Rsp3 blocks the antifungal activity of mannose-binding maize proteins

Lay-Sun Ma, Lei Wang, Christine Trippel, Artemio Mendoza-Mendoza, Steffen Ullmann, Marino Moretti, Alexander Carsten, Jörg Kahnt, Stefanie Reissmann, Bernd Zechmann, Gert Bange, and Regine Kahmann*

Supporting information

Supplementary Figures 1-13 and Supplementary Figure Legends

Supplementary Table 1- Visualization of Rsp3-HA by immunogold labeling in infected maize tissue

Supplementary Table 2- Plasmids and primers used in this study

Supplementary Table 3- *U. maydis* strains used in this study

Supplementary References

a


```
                *         20         *         40         *         60         *         80         *        100
FB1 : ATGAAGTTCCAAGCCTCGTTCGTGACCCTTCTGGTCGTCGTCAGCGCCGCCATCTCGGTAGCTGACGCCAAGGCCCCCATGGCCAACAAGCGACCATCCA : 100
FB2 : ATGAAGTTCCAAGCCTCGTTCGTGACCCTTCTGGTCGTCGTCAGCGCCGCCATCTCGGTAGCTGACGCCAAGGCCCCCATGGCCAACAAGCGACCATCCA : 100
I5  : ATGAAGTTCCAAGCCTCGTTCGTGACCCTTCTGGTCGTCGTCAGCGCCGCCATCTCGGTAGCTGACGCCAAGGCCCCCATGGCCAACAAGCGACCATCCA : 100
O3  : ATGAAGTTCCAAGCCTCGTTCGTGACCCTTCTGGTCGTCGTCAGCGCCGCCATCTCGGTAGCTGACGCCAAGGCCCCCATGGCCAACAAGCGACCATCCA : 100
P2  : ATGAAGTTCCAAGCCTCGTTCGTGACCCTTCTGGTCGTCGTCAGCGCCGCCATCTCGGTAGCTGACGCCAAGGCCCCCATGGCCAACAAGCGACCATCCA : 100
S3  : ATGAAGTTCCAAGCCTCGTTCGTGACCCTTCTGGTCGTCGTCAGCGCCGCCATCTCGGTAGCTGACGCCAAGGCCCCCATGGCCAACAAGCGACCATCCA : 100
T6  : ATGAAGTTCCAAGCCTCGTTCGTGACCCTTCTGGTCGTCGTCAGCGCCGCCATCTCGGTAGCTGACGCCAAGGCCCCCATGGCCAACAAGCGACCATCCA : 100

                *        120         *        140         *        160         *        180         *        200
FB1 : ATCTCAACCGCCGTGATGAGGCTGCCGCTCTCCAGAAGCGTGGTAGTCTCGGTGGCTTCGCCAATCGCTTCTTCCGCCGTGATGGTGGCGCTTATGGCGA : 200
FB2 : ATCTCAACCGCCGTGATGAGGCTGCCGCTCTCCAGAAGCGTGGTAGTCTCGGTGGCTTCGCCAATCGCTTCTTCCGCCGTGATGGTGGCGCTTATGGCGA : 200
I5  : ATCTCAACCGCCGTGATGAGGCTGCCGCTCTCCAGAAGCGTGGTAGTCTCGGTGGCTTCGCCAATCGCTTCTTCCGCCGTGATGGTGGCGCTTATGGCGA : 200
O3  : ATCTCAACCGCCGTGATGAGGCTGCCGCTCTCCAGAAGCGTGGTAGTCTCGGTGGCTTCGCCAATCGCTTCTTCCGCCGTGATGGTGGCGCTTATGGCGA : 200
P2  : ATCTCAACCGCCGTGATGAGGCTGCCGCTCTCCAGAAGCGTGGTAGTCTCGGTGGCTTCGCCAATCGCTTCTTCCGCCGTGATGGTGGCGCTTATGGCGA : 200
S3  : ATCTCAACCGCCGTGATGAGGCTGCCGCTCTCCAGAAGCGTGGTAGTCTCGGTGGCTTCGCCAATCGCTTCTTCCGCCGTGATGGTGGCGCTTATGGCGA : 200
T6  : ATCTCAACCGCCGTGATGAGGCTGCCGCTCTCCAGAAGCGTGGTAGTCTCGGTGGCTTCGCCAATCGCTTCTTCCGCCGTGATGGTGGCGCTTATGGCGA : 200

                *        220         *        240         *        260         *        280         *        300
FB1 : CAACCAGGACGCGGATGCTGGAGGTGACACCGCGAACCCTGACAGTCAAAACGGCAACCAGAGTGGTGACCAGCCTCCTCCCCCTGACACTGACGATGGC : 300
FB2 : CAACCAGGACGCGGATGCTGGAGGTGACACCGCGAACCCTGACAGTCAAAACGGCAACCAGAGTGGTGACCAGCCTCCTCCCCCTGACACTGACGATGGC : 300
I5  : CAACCAGGACGCGGATGCTGGAGGTGACACCGCGAACCCTGACAGTCAAAACGGCAACCAGAGTGGTGACCAGCCTCCTCCCCCTGACACTGACGATGGC : 300
O3  : CAACCAGGACGCGGATGCTGGAGGTGACACCGCGAACCCTGACAGTCAAAACGGCAACCAGAGTGGTGACCAGCCTCCTCCCCCTGACACTGACGATGGC : 300
P2  : CAACCAGGACGCGGATGCTGGAGGTGACACCGCGAACCCTGACAGTCAAAACGGCAACCAGAGTGGTGACCAGCCTCCTCCCCCTGACACTGACGATGGC : 300
S3  : CAACCAGGACGCGGATGCTGGAGGTGACACCGCGAACCCTGACAGTCAAAACGGCAACCAGAGTGGTGACCAGCCTCCTCCCCCTGACACTGACGATGGC : 300
T6  : CAACCAGGACGCGGATGCTGGAGGTGACACCGCGAACCCTGACAGTCAAAACGGCAACCAGAGTGGTGACCAGCCTCCTCCCCCTGACACTGACGATGGC : 300

                *        320         *        340         *        360         *        380         *        400
FB1 : AGCGTGCAACAGGGCTATGGCAATGACGAGCGACACCAAGGTCAGCCGCCTCCATCCCAACCCCAGCCTCAGCCTCAGCCCCAGCCCCAGCCCCAGCCCC : 400
FB2 : AGCGTGCAACAGGGCTATGGCAATGACGAGCGACACCAAGGTCAGCCGCCTCCATCCCAACCCCAGCCTCAGCCTCAGCCCCAGCCCCAGCCCCAGCCCC : 400
I5  : AACGTGCAACAGGGCTATGGCAATGACGAGCGACACCAAGGTCAGCCGCCTCCATCCCAACCCCAGCCTCAGCCTCAGCCCCAGCCCCAGCCCCAGCCCC : 400
O3  : AACGTGCAACAGGGCTATGGCAATGACGAGCGACACCAAGGTCAGCCGCCTCCATCCCAACCCCAGCCTCAGCCTCAGCCCCAGCCCCAGCCCCAGCCCC : 400
P2  : AACGTGCAACAGGGCTATGGCAATGACGAGCGACACCAAGGTCAGCCGCCTCCATCCCAACCCCAGCCTCAGCCTCAGCCCCAGCCCCAGCCCCAGCCCC : 400
S3  : AACGTGCAACAGGGCTATGGCAATGACGAGCGACACCAAGGTCAGCCGCCTCCATCCCAACCCCAGCCTCAGCCTCAGCCCCAGCCCCAGCCCCAGCCCC : 400
T6  : AACGTGCAACAGGGCTATGGCAATGACGAGCGACACCAAGGTCAGCCGCCTCCATCCCAACCCCAGCCTCAGCCTCAGCCCCAGCCCCAGCCCCAGCCCC : 400

                *        420         *        440         *        460         *        480         *        500
FB1 : AAACTCAACCTCAACCTCAGCCCCAGCCTCAACCTCAGGGTCAGGACTCGCAGCAGCAGCCAAAGACCGATCAGCAGAAGAAGGACGAGGAAAAGCAAAA : 500
FB2 : AAACTCAACCTCAACCTCAGCCCCAGCCTCAACCTCAGGGTCAGGACTCGCAGCAGCAGCCAAAGACCGATCAGCAGAAGAAGGACGAGGAAAAGCAAAA : 500
I5  : AAACTCAACCTCAACCTCAGCCCCAGCCTCAACCTCAGGGTCGGGACTCGCAGCAGCAGCCAAAGACCGATCAGCAGAAGAAGGACGAGGAAAAGCAAAA : 500
O3  : AAACTCAACCTCAACCTCAGCCCCAGCCTCAACCTCAGGGTCGGGACTCGCAGCAGCAGCCAAAGACCGATCAGCAGAAGAAGGACGAGGAAAAGCAAAA : 500
P2  : AAACTCAACCTCAACCTCAGCCCCAGCCTCAACCTCAGGGTCGGGACTCGCAGCAGCAGCCAAAGACCGATCAGCAGAAGAAGGACGAGGAAAAGCAAAA : 500
S3  : AAACTCAACCTCAACCTCAGCCCCAGCCTCAACCTCAGGGTCGGGACTCGCAGCAGCAGCCAAAGACCGATCAGCAGAAGAAGGACGAGGAAAAGCAAAA : 500
T6  : AAACTCAACCTCAACCTCAGCCCCAGCCTCAACCTCAGGGTCGGGACTCGCAGCAGCAGCCAAAGACCGATCAGCAGAAGAAGGACGAGGAAAAGCAAAA : 500

                *        520         *        540         *        560         *        580         *        600
FB1 : GCAAGACGAGCAGAAGCGTCAACAGGAGGAAGCGCAGCGACAGCAGAAGGAGCAGGAGCGTCAAAAGAAGGACGAAGAGAAGGCCAAGCAGCAGTCTCAG : 600
FB2 : GCAAGACGAGCAGAAGCGTCAACAGGAGGAAGCGCAGCGACAGCAGAAGGAGCAGGAGCGTCAAAAGAAGGACGAAGAGAAGGCCAAGCAGCAGTCTCAG : 600
I5  : GCAAGACGAGCAGAAGCGTCAACAGGAGGAAGCGCAGCGACAGCAGAAGGAGCAGGAGCGTCAAAAGAAGGACGAAGAGAAGGCCAAGCAGCAGTCTCAG : 600
O3  : GCAAGACGAGCAGAAGCGTCAACAGGAGGAAGCGCAGCGACAGCAGAAGGAGCAGGAGCGTCAAAAGAAGGACGAAGAGAAGGCCAAGCAGCAGTCTCAG : 600
P2  : GCAAGACGAGCAGAAGCGTCAACAGGAGGAAGCGCAGCGACAGCAGAAGGAGCAGGAGCGTCAAAAGAAGGACGAAGAGAAGGCCAAGCAGCAGTCTCAG : 600
S3  : GCAAGACGAGCAGAAGCGTCAACAGGAGGAAGCGCAGCGACAGCAGAAGGAGCAGGAGCGTCAAAAGAAGGACGAAGAGAAGGCCAAGCAGCAGTCTCAG : 600
T6  : GCAAGACGAGCAGAAGCGTCAACAGGAGGAAGCGCAGCGACAGCAGAAGGAGCAGGAGCGTCAAAAGAAGGACGAAGAGAAGGCCAAGCAGCAGTCTCAG : 600

                *        620         *        640         *        660         *        680         *        700
FB1 : GGCGGCAGCCAGAACGGTAACAACCAGCCTATTGGCTCTGGCAACGGTGGCCAGCCCGTTGCTCCTAACCAGAACCAGGAGACCACTATCCCTGACAACA : 700
FB2 : GGCGGCAGCCAGAACGGTAACAACCAGCCTATTGGCTCTGGCAACGGTGGCCAGCCCGTTGCTCCTAACCAGAACCAGGAGACCACTATCCCTGACAACA : 700
I5  : GGCGGCAGCCAGAACGGTAACAACCAGCCTATTGGCTCTGGCAACGGTGGCCAGCCCGTTGCTCCTAACCAGAACCAGGAGACCACTATCCCTGACAACA : 700
O3  : GGCGGCAGCCAGAACGGTAACAACCAGCCTATTGGCTCTGGCAACGGTGGCCAGCCCGTTGCTCCTAACCAGAACCAGGAGACCACTATCCCTGACAACA : 700
P2  : GGCGGCAGCCAGAACGGTAACAACCAGCCTATTGGCTCTGGCAACGGTGGCCAGCCCGTTGCTCCTAACCAGAACCAGGAGACCACTATCCCTGACAACA : 700
S3  : GGCGGCAGCCAGAACGGTAACAACCAGCCTATTGGCTCTGGCAACGGTGGCCAGCCCGTTGCTCCTAACCAGAACCAGGAGACCACTATCCCTGACAACA : 700
T6  : GGCGGCAGCCAGAACGGTAACAACCAGCCTATTGGCTCTGGCAACGGTGGCCAGCCCGTTGCTCCTAACCAGAACCAGGAGACCACTATCCCTGACAACA : 700

                *        720         *        740         *        760         *        780         *        800
FB1 : GCCAGGGTCAAGGCTCGGGTCCGGACAACTGCCCCAAGTTCCCTAAGAAGGGTGGTGGCGAAACTGATCATGCCCCATGCTTCTCCAAGGACGCCAACCG : 800
FB2 : GCCAGGGTCAAGGCTCGGGTCCGGACAACTGCCCCAAGTTCCCTAAGAAGGGTGGTGGCGAAACTGATCATGCCCCATGCTTCTCCAAGGACGCCAACCG : 800
I5  : GCCAGGGTCAAGGCTCGGGTCCGGACAACTGCCCCAAGTTCCCTAAGAAGGGTGGTGGCGAAACTGATCATGCCCCATGCTTCTCCAAGGACGCCAACCG : 800
O3  : GCCAGGGTCAAGGCTCGGGTCCGGACAACTGCCCCAAGTTCCCTAAGAAGGGTGGTGGCGAAACTGATCATGCCCCATGCTTCTCCAAGGACGCCAACCG : 800
P2  : GCCAGGGTCAAGGCTCGGGTCCGGACAACTGCCCCAAGTTCCCTAAGAAGGGTGGTGGCGAAACTGATCATGCCCCATGCTTCTCCAAGGACGCCAACCG : 800
S3  : GCCAGGGTCAAGGCTCGGGTCCGGACAACTGCCCCAAGTTCCCTAAGAAGGGTGGTGGCGAAACTGATCATGCCCCATGCTTCTCCAAGGACGCCAACCG : 800
T6  : GCCAGGGTCAAGGCTCGGGTCCGGACAACTGCCCCAAGTTCCCTAAGAAGGGTGGTGGCGAAACTGATCATGCCCCATGCTTCTCCAAGGACGCCAACCG : 800

                *        820         *        840         *        860         *        880         *        900
FB1 : CAAGGACATCTGCTCTGCTATGAAGCAGTTCGGTATTGATACCAAGGCCGTTGGGTGCTCCGACGACGAGTGCAGCGAGGACGCCAAGGACTTTTCGAAC : 900
FB2 : CAAGGACATCTGCTCTGCTATGAAGCAGTTCGGTATTGATACCAAGGCCGTTGGGTGCTCCGACGACGAGTGCAGCGAGGACGCCAAGGACTTTTCGAAC : 900
I5  : CAAGGACATCTGCTCTGCTATGAAGCAGTTCGGTATTGATACCAAGGCCGTTGGGTGCTCCGACGACGAGTGCAGCGAGGACGCCAAGGACTTTTCGAAC : 900
O3  : CAAGGACATCTGCTCTGCTATGAAGCAGTTCGGTATTGATACCAAGGCCGTTGGGTGCTCCGACGACGAGTGCAGCGAGGACGCCAAGGACTTTTCGAAC : 900
P2  : CAAGGACATCTGCTCTGCTATGAAGCAGTTCGGTATTGATACCAAGGCCGTTGGGTGCTCCGACGACGAGTGCAGCGAGGACGCCAAGGACTTTTCGAAC : 900
S3  : CAAGGACATCTGCTCTGCTATGAAGCAGTTCGGTATTGATACCAAGGCCGTTGGGTGCTCCGACGACGAGTGCAGCGAGGACGCCAAGGACTTTTCGAAC : 900
T6  : CAAGGACATCTGCTCTGCTATGAAGCAGTTCGGTATTGATACCAAGGCCGTTGGGTGCTCCGACGACGAGTGCAGCGAGGACGCCAAGGACTTTTCGAAC : 900
```

Supplementary Figure 1

```
               *       920         *       940         *       960         *       980         *      1000
FB1 : CACGCTTGCAACAAGAAGAAGCAGTGCGACTTCAACTGGGACAACAGCTGTGACTTCTACCTTCACATGGGCTTCACTAACGTCGCTAAGTTTGGCTGCG : 1000
FB2 : CACGCTTGCAACAAGAAGAAGCAGTGCGACTTCAACTGGGACAACAGCTGTGACTTCTACCTTCACATGGGCTTCACTAACGTCGCTAAGTTTGGCTGCG : 1000
I5  : CACGCTTGCAACAAGAAGAAGCAGTGCGACTTCAACTGGGACAACAGCTGTGACTTCTACCTTCACATGGGCTTCACTAACGTCGCTAAGTTTGGCTGCG : 1000
O3  : CACGCTTGCAACAAGAAGAAGCAGTGCGACTTCAACTGGGACAACAGCTGTGACTTCTACCTTCACATGGGCTTCACTAACGTCGCTAAGTTTGGCTGCG : 1000
P2  : CACGCTTGCAACAAGAAGAAGCAGTGCGACTTCAACTGGGACAACAGCTGTGACTTCTACCTTCACATGGGCTTCACTAACGTCGCTAAGTTTGGCTGCG : 1000
S3  : CACGCTTGCAACAAGAAGAAGCAGTGCGACTTCAACTGGGACAACAGCTGTGACTTCTACCTTCACATGGGCTTCACTAACGTCGCTAAGTTTGGCTGCG : 1000
T6  : CACGCTTGCAACAAGAAGAAGCAGTGCGACTTCAACTGGGACAACAGCTGTGACTTCTACCTTCACATGGGCTTCACTAACGTCGCTAAGTTTGGCTGCG : 1000

               *      1020         *      1040         *      1060         *      1080         *      1100
FB1 : GTGCCCCTAATGGCGGTGACGACGGTGACTCGTCTCTTCCCAGCGACGATAGCTCTGCTGGCCAGCAGCCTAGAGATAACAACCCTGGCCAGCAGCCCGG : 1100
FB2 : GTGCCCCTAATGGCGGTGACGACGGTGACTCGTCTCTTCCCAGCGACGATAGCTCTGCTGGCCAGCAGCCTAGAGATAACAACCCTGGCCAGCAGCCCGG : 1100
I5  : GTGCCCCTAATGGCGGTGACGACGGTGATTCGTCTCTTCCCAGCGACGATAGCTCTGCTGGCCAGCAGCCTAGAGACAACAACCCTGGCCAGCAGCCCGG : 1100
O3  : GTGCCCCTAATGGCGGTGACGACGGTGATTCGTCTCTTCCCAGCGACGATAGCTCTGCTGGCCAGCAGCCTAGAGACAACAACCCTGGCCAGCAGCCCGG : 1100
P2  : GTGCCCCTAATGGCGGTGACGACGGTGATTCGTCTCTTCCCAGCGACGATAGCTCTGCTGGCCAGCAGCCTAGAGACAACAACCCTGGCCAGCAGCCCGG : 1100
S3  : GTGCCCCTAATGGCGGTGACGACGGTGATTCGTCTCTTCCCAGCGACGATAGCTCTGCTGGCCAGCAGCCTAGAGACAACAACCCTGGCCAGCAGCCCGG : 1100
T6  : GTGCCCCTAATGGCGGTGACGACGGTGATTCGTCTCTTCCCAGCGACGATAGCTCTGCTGGCCAGCAGCCTAGAGACAACAACCCTGGCCAGCAGCCCGG : 1100

               *      1120         *      1140         *      1160         *      1180         *      1200
FB1 : AAACAACAACCCTGGTCAGCAGCCCGGAAAC---------------------------AACAACCCTGGTCAGCAGCCCGGAGACAACAACCCTGGTCAG : 1173
FB2 : AAACAACAACCCTGGTCAGCAGCCCGGAAACAACAACCCTGGTCAGCAGCCCGGAAACAACAACCCTGGTCAGCAGCCCGGAGACAACAACCCTGGTCAG : 1200
I5  : AAACAACAACCCTGGTCAGCAGCCCGGAAACAACAACCCTGGCCAGCAGCCCGGAAACAACAACCCTGGTCAGCAGCCCGGAGACAACAACCCTGGTCAG : 1200
O3  : AAACAACAACCCTGGT------------------------------------------------------CAGCAGCCCGGAGACAACAACCCTGGTCAG : 1146
P2  : AAACAACAACCCTGGTCAGCAGCCCGGAAACAACAACCCTGGCCAGCAGCCCGGAAACAACAACCCTGGTCAGCAGCCCGGAGACAACAACCCTGGTCAG : 1200
S3  : AAACAACAACCCTGGT------------------------------------------------------CAGCAGCCCGGAGACAACAACCCTGGTCAG : 1146
T6  : AAACAACAACCCTGGT------------------------------------------------------CAGCAGCCCGGAGACAACAACCCTGGTCAG : 1146

               *      1220         *      1240         *      1260         *      1280         *      1300
FB1 : CAAGCCGGCAAGGACGCCAACGAAGACGAGGAGTGCGACGATGAGGGTGACGACAAGGGTGACAACGGTGCTGCTGGCGGTGACAAGAACCCCAACGGTG : 1273
FB2 : CAAGCCGGCAAGGACGCCAACGAAGACGAGGAGTGCGACGATGAGGGTGACGACAAGGGTGACAACGGTGCTGCTGGCGGTGACAAGAACCCCAACGGTG : 1300
I5  : CAAGCCGGCAAGGACGCCAACGAAGACGAGGAGTGCGACGATGAGGGTGACGACAAGGGTGACAACGGTGCTGCTGGTGGTGACAAGAACCCCAACGGTG : 1300
O3  : CAAGCCGGCAAGGACGCCAACGAAGACGAGGAGTGCGACGATGAGGGTGACGACAAGGGTGACAACGGTGCTGCTGGTGGTGACAAGAACCCCAACGGTG : 1246
P2  : CAAGCCGGCAAGGACGCCAACGAAGACGAGGAGTGCGACGATGAGGGTGACGACAAGGGTGACAACGGTGCTGCTGGTGGTGACAAGAACCCCAACGGTG : 1300
S3  : CAAGCCGGCAAGGACGCCAACGAAGACGAGGAGTGCGACGATGAGGGTGACGACAAGGGTGACAACGGTGCTGCTGGTGGTGACAAGAACCCCAACGGTG : 1246
T6  : CAAGCCGGCAAGGACGCCAACGAAGACGAGGAGTGCGACGATGAGGGTGACGACAAGGGTGACAACGGTGCTGCTGGTGGTGACAAGAACCCCAACGGTG : 1246

               *      1320         *      1340         *      1360         *      1380         *      1400
FB1 : ACAACGGTGCTGCTGGCGGTGACAAGAACCCCAACGGTGACAACTCTTCTGAAGGTGACGAGCCGGTACCTTACGGTGACGAGACTGGTGACGCCGACAA : 1373
FB2 : ACAACGGTGCTGCTGGCGGTGACAAGAACCCCAACGGTGACAACTCTTCTGAAGGTGACGAGCCGGTACCTTACGGTGACGAGACTGGTGACGCCGACAA : 1400
I5  : ACAACGGTGCTGCCGGAGGTGACAAGAACCCCAACGGCGACAACGGTGCTGCCGGAGGTGACAAGAACCCCAACGGTGACAACTCTTCTGAAGGTGACGA : 1400
O3  : ACAACGGTGCTGCCGGAGGTGACAAGAACCCCAACGGCGACAACGGTGCTGCCGGAGGTGACAAGAACCCCAACGGTGACAACTCTTCTGAAGGTGACGA : 1346
P2  : ACAACGGTGCTGCCGGAGGTGACAAGAACCCCAACGGCGACAACGGTGCTGCCGGAGGTGACAAGAACCCCAACGGTGACAACTCTTCTGAAGGTGACGA : 1400
S3  : ACAACGGTGCTGCCGGAGGTGACAAGAACCCCAACGGTGACAACGGTGCTGCCGGAGGTGACAAGAACCCCAACGGCGA--------------------- : 1325
T6  : ACAACGGTGCTGCCGGAGGTGACAAGAACCCCAACGGCGACAACGGTGCTGCCGGAGGTGACAAGAACCCCAACGGTGACAACTCTTCTGAAGGTGACGA : 1346

               *      1420         *      1440         *      1460         *      1480         *      1500
FB1 : CGGTGCTGCTGGCGGTGACAAGAACCCCAGCGGTGACAAGAACCCCAACGGTGACAAGAACCCCAGCGGTGACAAGAACCCCAACGGTGACAACGGTGCT : 1473
FB2 : CGGTGCTGCTGGCGGTGACAAGAACCCCAGCGGTGACAAGAACCCCAACGG------------------------------------TGACAACGGTGCT : 1464
I5  : GCCGGTACCTTACGGTGACGAGACTGGTGACGCCGA---------------CAACGCTGCTGCTGGCGGTGACAAGAACCCCAACGGTGACAACTCTTCT : 1485
O3  : GCCGGTACCTTACGGTGACGAGACTGGTGACGCCGA---------------CAACGCTGCTGCTGGCGGTGACAAGAACCCCAACGGTGACAACTCTTCT : 1431
P2  : GCCGGTACCTTACGGTGACGAGACTGGTGACGCCGA---------------CAACGCTGCTGCTGGCGGTGACAAGAACCCCAACGGTGACAACTCTTCT : 1485
S3  : ---------------------------------------------------CAACGGTGCTGCCGGAGGTGACAAGAACCCCAACGGTGACAACTCTTCT : 1374
T6  : GCCGGTACCTTACGGTGACGAGACTGGTGACGCCGA---------------CAACGCTGCTGCTGGCGGTGACAAGAACCCCAACGGTGACAACTCTTCT : 1431

               *      1520         *      1540         *      1560         *      1580         *      1600
FB1 : GCTGGTGGTGACAAGAACCCCAACGGTGACAACGGTGCTGCCGGAGGTGACAAGAACCCCAACGGTGACAACGGTGCTGCTGGCGGTGACAAGAACCCCA : 1573
FB2 : GCTGGTGGTGACAAGAACCCCAGCGGTGAC---------------------AAGAACCCCAACGGTGACAACGGTGCTGCC------------------- : 1524
I5  : GAAGGTGA---------------------CGAGCCGGTACCTTACGGTGACGAGACTGGTGACGCCGACAACGCTGCTGCTGGCGGTGACAAGAACCCCA : 1564
O3  : GAAGGTGA---------------------CGAGCCGGTACCTTACGGTGACGAGACTGGTGACGCCGACAACGCTGCTGCTGGCGGTGACAAGAACCCCA : 1510
P2  : GAAGGTGA---------------------CGAGCCGGTACCTTACGGTGACGAGACTGGTGACGCCGACAACGCTGCTGCTGGCGGTGACAAGAACCCCA : 1564
S3  : GAAGGTGA---------------------CGAGCCGGTACCTTACGGTGACGAGACTGGTGACGCCGACAACGCTGCTGCTGGCGGTGACAAGAACCCCA : 1453
T6  : GAAGGTGA---------------------CGAGCCG---------------------------------------------------------------- : 1446

               *      1620         *      1640         *      1660         *      1680         *      1700
FB1 : ACGGTGACAACTCTTCTGAAGGTGACGAGCCGGTACCTTACGGCGACGAGACTGGTGACGCCGACAACGGTGCTGCTGGCGGTGACAAGAACCCCAACGG : 1673
FB2 : --------------------------------------GGAGGTGACAAGAACCCCAACGGTGACAACGGTGCTGCTGGAGGTGACAAGAACCCCAACGG : 1586
I5  : ACGGTGACAACTCTTCTGAAGGCGACGAGCCGGTACCTTACGGTGACGAGACTGGTGACGCCGACAACGCTGCTGCTGGTGGTGACAAGAACCCCAGCGG : 1664
O3  : ACGGTGACAACTCTTCTGAAGGTGACGAGCCGGTACCTTACGGTGACGAGACTGGTGACGCCGACAACGCTGCTGCTGGCGGTGACAAGAACCCCAACGG : 1610
P2  : ACGGTGACAACTCTTCTGAAGGCGACGAGCCGGTACCTTACGGTGACGAGACTGGTGACGCCGACAACGCTGCTGCTGGTGGTGACAAGAACCCCAGCGG : 1664
S3  : ACGGTGACAACTCTTCTGAAGGTGACGAGCCGGTACCTTACGGTGACGAGACTGGTGACGCCGACAACGCTGCTGCTGGTGGTGACAAGAACCCCAGCGG : 1553
T6  : --------------------------------GTACCTTACGGTGACGAGACTGGTGACGCCGACAACGCTGCTGCTGGCGGTGACAAGAACCCCAACGG : 1514

               *      1720         *      1740         *      1760         *      1780         *      1800
FB1 : TGACAACTCTTCTGAAGGTGACGAGCCGGTACCTTACGGCGACGAGACTGGTGACGCCGACAACGGTGCTGCTGGCGGTGACAAGAACCCCAGTGGTGAC : 1773
FB2 : TGACAACTCTTCTGAAGGTGACGAGCCGGTACCTTACGGCGACGAGACTGGTGACGCCGACAACGGTGCTGCTGGCGGTGACAAGAACCCCAGTGGTGAC : 1686
I5  : T------------------------------------------------------------------------------GACAAGAAC------------ : 1674
O3  : TGACAACTCTTCTGAAGGTGACGAGCCGGTACCTTACGGTGACGAGACTGGTGACGCCGACAACGCTGCTGCTGGTGGTGACAAGAAC------------ : 1698
P2  : T------------------------------------------------------------------------------GACAAGAAC------------ : 1674
S3  : T------------------------------------------------------------------------------GACAAGAAC------------ : 1563
T6  : TGACAACTCTTCTGAAGGTGACGAGCCGGTACCTTACGGTGACGAGACTGGTGACGCCGACAACGCTGCTGCTGGTGGTGACAAGAAC------------ : 1602
```

Supplementary Figure 1

```
                  *        1820         *        1840         *        1860         *        1880         *        1900
FB1 : AAGAACCCCAACGGTGACAAGAACCCCAACGGTGACAACGGTGCTGCCGGAGGTGACAAGAACCCCAACGGTGACAACGGTGCTGCCGGAGGTGACAAGA : 1873
FB2 : AAGAACCCCAACGGTGACAAGAACCCCAACGGTGACAACGGTGCTGCCGGAGGTGACAAGAACCCCAACGGTGACAACGGTGCTGCTGGCGGTGACAAGA : 1786
I5  : ------------------------CCCAACGGTGACAACGGTGCTGCCGGAGGTGACAAGAACCCCAACGGTGACAACGGTGCTGCTGGCGGTGACAAGA : 1750
O3  : ------------------------CCCAACGGTGACAACGGTGCTGCCGGAGGTGACAAGAACCCCAACGGTGACAACGGTGCTGCTGGCGGTGACAAGA : 1774
P2  : ------------------------CCCAACGGTGACAACGGTGCTGCCGGAGGTGACAAGAACCCCAACGGTGACAACGGTGCTGCTGGCGGTGACAAGA : 1750
S3  : ------------------------CCCAACGGTGACAACGGTGCTGCCGGAGGTGACAAGAACCCCAACGGTGACAACGGTGCTGCTGGCGGTGACAAGA : 1639
T6  : ------------------------CCCAACGGTGACAACGGTGCTGCCGGAGGTGACAAGAACCCCAACGGTGACAACGGTGCTGCTGGCGGTGACAAGA : 1678

                  *        1920         *        1940         *        1960         *        1980         *        2000
FB1 : ACCCCAACGGTGACAACTCTTCTGAAGGTGACGAGCCGGTACCTTACGGCGACGAGACTGGTGACGCCGACAACGGTGCTGCTGGCGGTGACAAGAACCC : 1973
FB2 : ACCCCAACGGTGACAACTCTTCTGAAGGTGACGAGCCGGTACCTTACGGCGACGAGACTGGTGACGCCGACAACGGTGCTGCTGGCGGTGACAAGAACCC : 1886
I5  : ACCCCAACGGTGACAACTCTTCTGAAGGCGACGAGCCGGTACCTTACGGCGACGAGACTGGTGACGCCGACAACGCTGCTGCTGGTGACAAGGGCGCTAA : 1850
O3  : ACCCCAACGGTGACAACTCTTCTGAAGGCGACGAGCCGGTACCTTACGGCGACGAGACTGGTGACGCCGACAACGCTGCTGCTGGTGACAAGGGCGCTAA : 1874
P2  : ACCCCAACGGTGACAACTCTTCTGAAGGCGACGAGCCGGTACCTTACGGCGACGAGACTGGTGACGCCGACAACGCTGCTGCTGGTGACAAGGGCGCTAA : 1850
S3  : ACCCCAACGGTGACAACTCTTCTGAAGGCGACGAGCCGGTACCTTACGGCGACGAGACTGGTGACGCCGACAACGCTGCTGCTGGTGACAAGGGCGCTAA : 1739
T6  : ACCCCAACGGTGACAACTCTTCTGAAGGCGACGAGCCGGTACCTTACGGCGACGAGACTGGTGACGCCGACAACGCTGCTGCTGGTGACAAGGGCGCTAA : 1778

                  *        2020         *        2040         *        2060         *        2080         *        2100
FB1 : CAGCGGTGACAAGAACCCCAACGGTGACAAGAACCCCAGCGGTGACAAGAACCCCAACGGTGACAACGGTGCTGCTGGTGGTGACAAGAACCCCAGCGGT : 2073
FB2 : CAGTGGTGACAAGAAC---------------------------------------------------------------------------------- : 1902
I5  : CAAGCCCGACGATTGCAAGAAGAACAAGGATGCGTCTGCCGGTGGTGA---CACCCAGGACTCGAAGGATGCTCAGGGCGACTCCAAGGCCCCGCAGCCT : 1947
O3  : CAAGCCCGACGATTGCAAGAAGAACAAGGATGCGTCTGCCGGTGGTGA---CACCCAGGACTCGAAGGATGCTCAGGGCGACTCCAAGGCCCCGCAGCCT : 1971
P2  : CAAGCCCGACGATTGCAAGAAGAACAAGGATGCGTCTGCCGGTGGTGA---CACCCAGGACTCGAAGGATGCTCAGGGCGACTCCAAGGCCCCGCAGCCT : 1947
S3  : CAAGCCCGACGATTGCAAGAAGAACAAGGATGCGTCTGCCGGTGGTGA---CACCCAGGACTCGAAGGATGCTCAGGGCGACTCCAAGGCCCCGCAGCCT : 1836
T6  : CAAGCCCGACGATTGCAAGAAGAACAAGGATGCGTCTGCCGGTGGTGA---CACCCAGGACTCGAAGGATGCTCAGGGCGACTCCAAGGCCCCGCAGCCT : 1875

                  *        2120         *        2140         *        2160         *        2180         *        2200
FB1 : GACAAGAACCCCAACGGTGACAACGGTGCTGCCGGAGGTGACAAGAACCCCAACGGTGACAACGGTGCTGCCGGAGGTGACAAGAACCCCAACGGTGACA : 2173
FB2 : ---------------------------------------------------------------------CCCAACGGTGACAAGAACCCCAACGGTGACA : 1933
I5  : TAG------------------------------------------------------------------------------------------------- : 1950
O3  : TAG------------------------------------------------------------------------------------------------- : 1974
P2  : TAG------------------------------------------------------------------------------------------------- : 1950
S3  : TAG------------------------------------------------------------------------------------------------- : 1839
T6  : TAG------------------------------------------------------------------------------------------------- : 1878

                  *        2220         *        2240         *        2260         *        2280         *        2300
FB1 : ACGGTGCTGCCGGAGGTGACAAGAACCCCAACGGTGACAACGGTGCTGCTGGCGGTGACAAGAACCCCAACGGTGACAACTCTTCTGAAGGTGACGAGCC : 2273
FB2 : ACGGTGCTGCCGGAGGTGACAAGAACCCCAACGGTGACAACGGTGCTGCTGGCGGTGACAAGAACCCCAACGGTGACAACTCTTCTGAAGGTGACGAGCC : 2033
I5  : ---------------------------------------------------------------------------------------------------- :    -
O3  : ---------------------------------------------------------------------------------------------------- :    -
P2  : ---------------------------------------------------------------------------------------------------- :    -
S3  : ---------------------------------------------------------------------------------------------------- :    -
T6  : ---------------------------------------------------------------------------------------------------- :    -

                  *        2320         *        2340         *        2360         *        2380         *        2400
FB1 : GGTACCTTACGGCGACGAGACTGGTGACGCCGACAACGGTGCTGCTGGTGGTGACAAGAACCCCAGCGGTGACAACGGTGCTGCCGGAGGTGACAAGAAC : 2373
FB2 : GGTACCTTACGGCGACGAGACTGGTGACGCCGACAACGGTGCTGCTGGTGGTGACAAGAACCCCAGCGGTGACAACGGTGCTGCCGGAGGTGACAAGAAC : 2133
I5  : ---------------------------------------------------------------------------------------------------- :    -
O3  : ---------------------------------------------------------------------------------------------------- :    -
P2  : ---------------------------------------------------------------------------------------------------- :    -
S3  : ---------------------------------------------------------------------------------------------------- :    -
T6  : ---------------------------------------------------------------------------------------------------- :    -

                  *        2420         *        2440         *        2460         *        2480         *        2500
FB1 : CCCAACGGTGACAACGGTGCTGCCGGAGGTGACAAGAACCCCAACGGCGACAACTCTTCTGAAGGCGACGAGCCGGTACCTTACGGCGACGAGACTGGCG : 2473
FB2 : CCCAACGGTGACAACGGTGCTGCCGGAGGTGACAAGAACCCCAACGGCGACAACTCTTCTGAAGGCGACGAGCCGGTACCTTACGGCGACGAGACTGGCG : 2233
I5  : ---------------------------------------------------------------------------------------------------- :    -
O3  : ---------------------------------------------------------------------------------------------------- :    -
P2  : ---------------------------------------------------------------------------------------------------- :    -
S3  : ---------------------------------------------------------------------------------------------------- :    -
T6  : ---------------------------------------------------------------------------------------------------- :    -

                  *        2520         *        2540         *        2560         *        2580         *        2600
FB1 : ACGCCGACAACGGTGCTGCTGGTGACAAGGGCGCTAACAAGCCCGACGATTGCAAGAAGAACAAGGATGCGTCTGCCGGTGGTGACACCCAGGACTCGAA : 2573
FB2 : ACGCCGACAACGGTGCTGCTGGTGACAAGGGCGCTAACAAGCCCGACGATTGCAAGAAGAACAAGGATGCGTCTGCCGGTGGTGACACCCAGGACTCGAA : 2333
I5  : ---------------------------------------------------------------------------------------------------- :    -
O3  : ---------------------------------------------------------------------------------------------------- :    -
P2  : ---------------------------------------------------------------------------------------------------- :    -
S3  : ---------------------------------------------------------------------------------------------------- :    -
T6  : ---------------------------------------------------------------------------------------------------- :    -

                  *        2620         *
FB1 : GGATGCTCAGGGCGACTCCAAGGCCCCGCAGCCTTAG : 2610
FB2 : GGATGCTCAGGGCGACTCCAAGGCCCCGCAGCCTTAG : 2370
I5  : ------------------------------------- :    -
O3  : ------------------------------------- :    -
P2  : ------------------------------------- :    -
S3  : ------------------------------------- :    -
T6  : ------------------------------------- :    -
```

b

Supplementary Figure 1

```
              *        20         *        40         *        60         *        80         *       100
FB1 : MKFQASFVTLLVVVSAAISVADAKAPMANKRPSNLNRRDEAAALQKRGSLGGFANRFFRRDGGAYGDNQDADAGGDTANPDSQNGNQSGDQPPPPDTDDG : 100
FB2 : MKFQASFVTLLVVVSAAISVADAKAPMANKRPSNLNRRDEAAALQKRGSLGGFANRFFRRDGGAYGDNQDADAGGDTANPDSQNGNQSGDQPPPPDTDDG : 100
I5  : MKFQASFVTLLVVVSAAISVADAKAPMANKRPSNLNRRDEAAALQKRGSLGGFANRFFRRDGGAYGDNQDADAGGDTANPDSQNGNQSGDQPPPPDTDDG : 100
O3  : MKFQASFVTLLVVVSAAISVADAKAPMANKRPSNLNRRDEAAALQKRGSLGGFANRFFRRDGGAYGDNQDADAGGDTANPDSQNGNQSGDQPPPPDTDDG : 100
P2  : MKFQASFVTLLVVVSAAISVADAKAPMANKRPSNLNRRDEAAALQKRGSLGGFANRFFRRDGGAYGDNQDADAGGDTANPDSQNGNQSGDQPPPPDTDDG : 100
S3  : MKFQASFVTLLVVVSAAISVADAKAPMANKRPSNLNRRDEAAALQKRGSLGGFANRFFRRDGGAYGDNQDADAGGDTANPDSQNGNQSGDQPPPPDTDDG : 100
T6  : MKFQASFVTLLVVVSAAISVADAKAPMANKRPSNLNRRDEAAALQKRGSLGGFANRFFRRDGGAYGDNQDADAGGDTANPDSQNGNQSGDQPPPPDTDDG : 100
      MKFQASFVTLLVVVSAAISVADAKAPMANKRPSNLNRRDEAAALQKRGSLGGFANRFFRRDGGAYGDNQDADAGGDTANPDSQNGNQSGDQPPPPDTDDG

              *       120         *       140         *       160         *       180         *       200
FB1 : SVQQGYGNDERHQGQPPPSQPQPQPQPQPQPQPQTQPQPQPQPQPQGQDSQQQPKTDQQKKDEEKQKQDEQKRQQEEAQRQQKEQERQKKDEEKAKQQSQ : 200
FB2 : SVQQGYGNDERHQGQPPPSQPQPQPQPQPQPQPQTQPQPQPQPQPQGQDSQQQPKTDQQKKDEEKQKQDEQKRQQEEAQRQQKEQERQKKDEEKAKQQSQ : 200
I5  : NVQQGYGNDERHQGQPPPSQPQPQPQPQPQPQPQTQPQPQPQPQPQGRDSQQQPKTDQQKKDEEKQKQDEQKRQQEEAQRQQKEQERQKKDEEKAKQQSQ : 200
O3  : NVQQGYGNDERHQGQPPPSQPQPQPQPQPQPQPQTQPQPQPQPQPQGRDSQQQPKTDQQKKDEEKQKQDEQKRQQEEAQRQQKEQERQKKDEEKAKQQSQ : 200
P2  : NVQQGYGNDERHQGQPPPSQPQPQPQPQPQPQPQTQPQPQPQPQPQGRDSQQQPKTDQQKKDEEKQKQDEQKRQQEEAQRQQKEQERQKKDEEKAKQQSQ : 200
S3  : NVQQGYGNDERHQGQPPPSQPQPQPQPQPQPQPQTQPQPQPQPQPQGRDSQQQPKTDQQKKDEEKQKQDEQKRQQEEAQRQQKEQERQKKDEEKAKQQSQ : 200
T6  : NVQQGYGNDERHQGQPPPSQPQPQPQPQPQPQPQTQPQPQPQPQPQGRDSQQQPKTDQQKKDEEKQKQDEQKRQQEEAQRQQKEQERQKKDEEKAKQQSQ : 200
       VQQGYGNDERHQGQPPPSQPQPQPQPQPQPQPQTQPQPQPQPQPQG DSQQQPKTDQQKKDEEKQKQDEQKRQQEEAQRQQKEQERQKKDEEKAKQQSQ

              *       220         *       240         *       260         *       280         *       300
FB1 : GGSQNGNNQPIGSGNGGQPVAPNQNQETTIPDNSQGQGSGPDNCPKFPKKGGGETDHAPCFSKDANRKDICSAMKQFGIDTKAVGCSDDECSEDAKDFSN : 300
FB2 : GGSQNGNNQPIGSGNGGQPVAPNQNQETTIPDNSQGQGSGPDNCPKFPKKGGGETDHAPCFSKDANRKDICSAMKQFGIDTKAVGCSDDECSEDAKDFSN : 300
I5  : GGSQNGNNQPIGSGNGGQPVAPNQNQETTIPDNSQGQGSGPDNCPKFPKKGGGETDHAPCFSKDANRKDICSAMKQFGIDTKAVGCSDDECSEDAKDFSN : 300
O3  : GGSQNGNNQPIGSGNGGQPVAPNQNQETTIPDNSQGQGSGPDNCPKFPKKGGGETDHAPCFSKDANRKDICSAMKQFGIDTKAVGCSDDECSEDAKDFSN : 300
P2  : GGSQNGNNQPIGSGNGGQPVAPNQNQETTIPDNSQGQGSGPDNCPKFPKKGGGETDHAPCFSKDANRKDICSAMKQFGIDTKAVGCSDDECSEDAKDFSN : 300
S3  : GGSQNGNNQPIGSGNGGQPVAPNQNQETTIPDNSQGQGSGPDNCPKFPKKGGGETDHAPCFSKDANRKDICSAMKQFGIDTKAVGCSDDECSEDAKDFSN : 300
T6  : GGSQNGNNQPIGSGNGGQPVAPNQNQETTIPDNSQGQGSGPDNCPKFPKKGGGETDHAPCFSKDANRKDICSAMKQFGIDTKAVGCSDDECSEDAKDFSN : 300
      GGSQNGNNQPIGSGNGGQPVAPNQNQETTIPDNSQGQGSGPDNCPKFPKKGGGETDHAPCFSKDANRKDICSAMKQFGIDTKAVGCSDDECSEDAKDFSN

              *       320         *       340         *       360         *       380         *       400
FB1 : HACNKKKQCDFNWDNSCDFYLHMGFTNVAKFGCGAPNGGDDGDSSLPSDDSSAGQQPRDNNPGQQPGNNNPGQQPG---------NNNPGQQPGDNNPGQ : 391
FB2 : HACNKKKQCDFNWDNSCDFYLHMGFTNVAKFGCGAPNGGDDGDSSLPSDDSSAGQQPRDNNPGQQPGNNNPGQQPGNNNPGQQPGNNNPGQQPGDNNPGQ : 400
I5  : HACNKKKQCDFNWDNSCDFYLHMGFTNVAKFGCGAPNGGDDGDSSLPSDDSSAGQQPRDNNPGQQPGNNNPGQQPGNNNPGQQPGNNNPGQQPGDNNPGQ : 400
O3  : HACNKKKQCDFNWDNSCDFYLHMGFTNVAKFGCGAPNGGDDGDSSLPSDDSSAGQQPRDNNPGQQPGNN------------------NPGQQPGDNNPGQ : 382
P2  : HACNKKKQCDFNWDNSCDFYLHMGFTNVAKFGCGAPNGGDDGDSSLPSDDSSAGQQPRDNNPGQQPGNNNPGQQPGNNNPGQQPGNNNPGQQPGDNNPGQ : 400
S3  : HACNKKKQCDFNWDNSCDFYLHMGFTNVAKFGCGAPNGGDDGDSSLPSDDSSAGQQPRDNNPGQQPGNNN------------------PGQQPGDNNPGQ : 382
T6  : HACNKKKQCDFNWDNSCDFYLHMGFTNVAKFGCGAPNGGDDGDSSLPSDDSSAGQQPRDNNPGQQPGNNN------------------PGQQPGDNNPGQ : 382
      HACNKKKQCDFNWDNSCDFYLHMGFTNVAKFGCGAPNGGDDGDSSLPSDDSSAGQQPRDNNPGQQPGNNn                  PGQQPGDNNPGQ

              *       420         *       440         *       460         *       480         *       500
FB1 : QAGKDANEDEECDDEGDDKGDNGAAGGDKNPNGDNGAAGGDKNPNGDNSSEGDEPVPYGDETGDADNGAAGGDKNPSGDKNPNGDKNPSGDKNPNGDNGA : 491
FB2 : QAGKDANEDEECDDEGDDKGDNGAAGGDKNPNGDNGAAGGDKNPNGDNSSEGDEPVPYGDETGDADNGAAGGDKNPSGDKNPNGDNGAAG------GDKN : 494
I5  : QAGKDANEDEECDDEGDDKGDNGAAGGDKNPNGDN----------------------------------------------------------------- : 435
O3  : QAGKDANEDEECDDEGDDKGDNGAAGGDKNPNGDN----------------------------------------------------------------- : 417
P2  : QAGKDANEDEECDDEGDDKGDNGAAGGDKNPNGDN----------------------------------------------------------------- : 435
S3  : QAGKDANEDEECDDEGDDKGDNGAAGGDKNPNGDN----------------------------------------------------------------- : 417
T6  : QAGKDANEDEECDDEGDDKGDNGAAGGDKNPNGDN----------------------------------------------------------------- : 417
      QAGKDANEDEECDDEGDDKGDNGAAGGDKNPNGDN

              *       520         *       540         *       560         *       580         *       600
FB1 : AGGDKNPNGDNGAAGGDKNPNGDNGAAGGDKNPNGDNSSEGDEPVPYGDETGDADNGAAGGDKNPNGDNSSEGDEPVPYGDETGDADNGAAGGDKNPSGD : 591
FB2 : PSGDKNPNGDNGAAGGDKNPNGDNGAAG--------------------------------GDKNPNGDNSSEGDEPVPYGDETGDADNGAAGGDKNPSGD : 562
I5  : -----------GAAGGDKNPNGDNGAAGGDKNPNGDNSSEGDEPVPYGDETGDADNAAAGGDKNPNGDNSSEGDEPVPYGDETGDADNAAAG-------- : 516
O3  : -----------GAAGGDKNPNGDNGAAGGDKNPNGDNSSEGDEPVPYGDETGDADNAAAGGDKNPNGDNSSEGDEPVPYGDETGDADNAAAG-------- : 498
P2  : -----------GAAGGDKNPNGDNGAAGGDKNPNGDNSSEGDEPVPYGDETGDADNAAAGGDKNPNGDNSSEGDEPVPYGDETGDADNAAAG-------- : 516
S3  : -----------GAAGGDKNPNGDNGAAGGDKNPNGDN-------------------GAAGGDKNPNGDNSSEGDEPVPYGDETGDADNAAAG-------- : 479
T6  : -----------GAAGGDKNPNGDNGAAGGDKNPNGDNSSEGDEPVPYGDETGDADNAAAGGDKNPNGDNSSEGDEPVPYGDETGDADNAAAG-------- : 498
                 GAAGGDKNPNGDNGAAGgdknpngdn                    aagGDKNPNGDNSSEGDEPVPYGDETGDADN AAG

              *       620         *       640         *       660         *       680         *       700
FB1 : KNPNGDKNPNGDNGAAGGDKNPNGDNGAAGGDKNPNGDNSSEGDEPVPYGDETGDADNGAAGGDKNPSGDKNPNGDKNPSGDKNPNGDNGAAGGDKNPSG : 691
FB2 : KNPNGDKNPNGDNGAAGGDKNPNGDNGAAGGDKNPNGDNSSEGDEPVPYGDETGDADNGAAGGDKNPSGDKNPNGDKN---------------------- : 640
I5  : ------------------------------GDKNPNGDNSSEGDEPVPYGDETGDADNAAAGGDKNPSGDKNPNG------------------------- : 561
O3  : ------------------------------GDKNPNGDNSSEGDEPVPYGDETGDADNAAAGGD                                     : 532
P2  : ------------------------------GDKNPNGDNSSEGDEPVPYGDETGDADNAAAGGDKNPSGDKNPNG------------------------- : 561
S3  : ------------------------------GDKNPNGDNSSEGDEPVPYGDETGDADNAAAGGDKNPSGDKNPNG------------------------- : 524
T6  : ------------------------------GDKNPNGDNSSEGDEPVPYGDETGDADNAAAGGDK------NPNG------------------------- : 537
                                    GDKNPNGDNSSEGDEPVPYGDETGDADN AAGGDk      npng

              *       720         *       740         *       760         *       780         *       800
FB1 : DKNPNGDNGAAGGDKNPNGDNGAAGGDKNPNGDNGAAGGDKNPNGDNGAAGGDKNPNGDNSSEGDEPVPYGDETGDADNGAAGGDKNPSGDNGAAGGDKN : 791
FB2 : -----------------------------PNGDNGAAGGDKNPNGDNGAAGGDKNPNGDNSSEGDEPVPYGDETGDADNGAAGGDKNPSGDNGAAGGDKN : 711
I5  : ------------------------------------------------------------------------------------------DNGAAGGDKN : 571
O3  : -----------------------------------------------------KNPNGDNSSEGDEPVPYGDETGDADNAAAGGDKNPNGDNGAAGGDKN : 579
P2  : ------------------------------------------------------------------------------------------DNGAAGGDKN : 571
S3  : ------------------------------------------------------------------------------------------DNGAAGGDKN : 534
T6  : ------------------------------------------------------------------------------------------DNGAAGGDKN : 547
                                                                                                DNGAAGGDKN

              *       820         *       840         *       860         *
FB1 : PNGDNGAAGGDKNPNGDNSSEGDEPVPYGDETGDADNGAAGDKGANKPDDCKKNKDASAGGDTQDSKDAQGDSKAPQP : 869
FB2 : PNGDNGAAGGDKNPNGDNSSEGDEPVPYGDETGDADNGAAGDKGANKPDDCKKNKDASAGGDTQDSKDAQGDSKAPQP : 789
I5  : PNGDNGAAGGDKNPNGDNSSEGDEPVPYGDETGDADNAAAGDKGANKPDDCKKNKDASAGGDTQDSKDAQGDSKAPQP : 649
O3  : PNGDNGAAGGDKNPNGDNSSEGDEPVPYGDETGDADNAAAGDKGANKPDDCKKNKDASAGGDTQDSKDAQGDSKAPQP : 657
P2  : PNGDNGAAGGDKNPNGDNSSEGDEPVPYGDETGDADNAAAGDKGANKPDDCKKNKDASAGGDTQDSKDAQGDSKAPQP : 649
S3  : PNGDNGAAGGDKNPNGDNSSEGDEPVPYGDETGDADNAAAGDKGANKPDDCKKNKDASAGGDTQDSKDAQGDSKAPQP : 612
T6  : PNGDNGAAGGDKNPNGDNSSEGDEPVPYGDETGDADNAAAGDKGANKPDDCKKNKDASAGGDTQDSKDAQGDSKAPQP : 625
      PNGDNGAAGGDKNPNGDNSSEGDEPVPYGDETGDADN AAGDKGANKPDDCKKNKDASAGGDTQDSKDAQGDSKAPQP
```

Supplementary Figure 1: Sequence alignment of *rsp3* alleles from different isolates. Genomic DNA from *U. maydis* strains FB1 and FB2 and Mexican isolates I5, O3, P2, S3 and T6 was extracted and used as template to amplify *rsp3* using primer pair Rsp3 #48/49. (**a**) The PCR products were sequenced and the nucleotide sequences were aligned. (**b**) The *rsp3* alleles depicted in (a) were translated to amino acid sequence using ExPASy Translate (http://web.expasy.org/translate/) and aligned.

Supplementary Figure 2

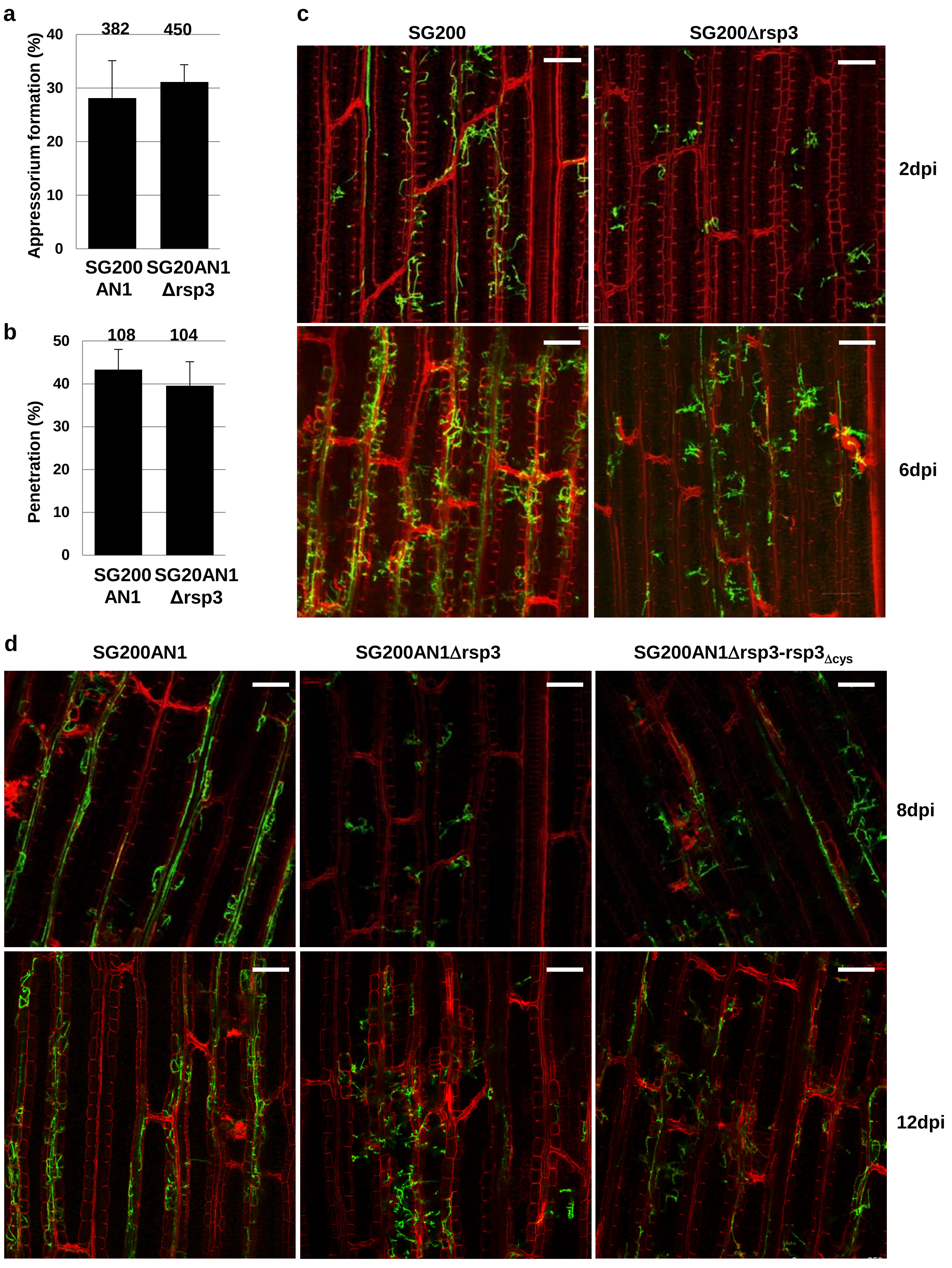

e

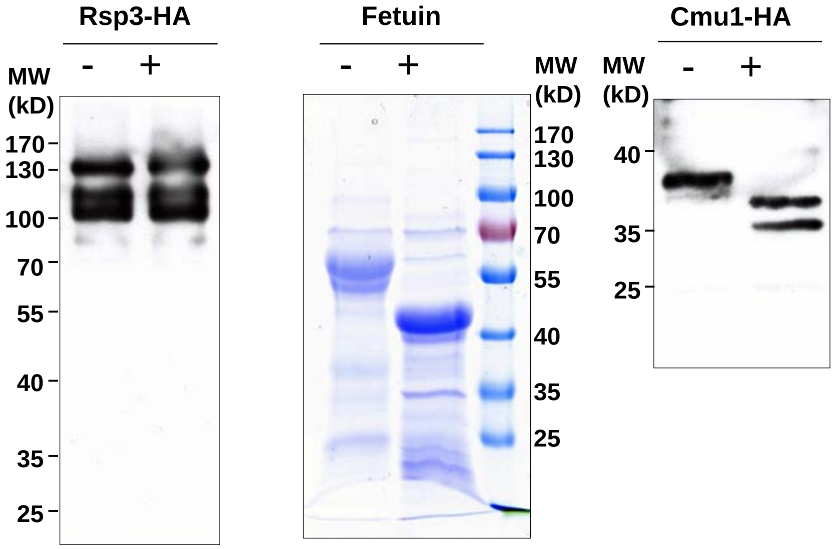

Supplementary Figure 2: Plant colonization by *rsp3* mutants and glycosylation status of Rsp3. (**a**) Seven-day-old maize seedlings were infected with SG200AN1 and SG200AN1Δrsp3. Appressoria formation was quantified with the help of eGFP fluorescence of the AN1 marker relative to filament formation 18 h post infection. Numbers of analyzed filaments are indicated above the respective columns. Error bars indicate standard deviations. Values represent mean ± s.d. of three replicates, evaluating at least 100 filaments per sample. (**b**) Penetration efficiency of the indicated strains was quantified 18 h post infection. The percentage of appressoria that had developed penetrating hyphae was determined relative to the total number of appressoria showing eGFP fluorescence. For each strain more than 100 appressoria were analyzed in three independent experiments. Numbers of analyzed appressoria are indicated above the respective columns. Error bars indicate standard deviations. Values represent mean ± s.d. of three replicates. (**c,d**) Maize seedlings infected by the indicated strains were observed at the indicated dpi by confocal microscopy. Fungal hyphae were stained with WGA-AF488 (green). Plant cell walls were stained with propidium iodide (red). Bars: 100 µm. (**e**) Rsp3 is not glycosylated. Rsp3-HA or Cmu1-HA was purified from culture supernatant of SG200Δrsp3-P_{otef}-rsp3-HA or SG200Δcmu1-P_{otef}-cmu1-HA respectively, using anti-HA agarose beads. The indicated proteins were treated with (+) or without (-) deglycosylation enzyme mix (containing PNGase F, *O*-glycosidase, β1-4 galactosidase, β-N-acetylglucosaminidase, and neuraminidase) according to the manufacturer´s protocol (NEB Cat# P6039). Fetuin (NEB Cat# P6039) which contains *N*- and *O*-linked glycans and Cmu1-HA served as positive controls. The protein samples were separated on SDS-PAGE and analyzed by western blotting using the anti-HA antibody (Rsp3-HA and Cmu1-HA) or coomassie-blue staining (fetuin).

Supplementary Figure 3

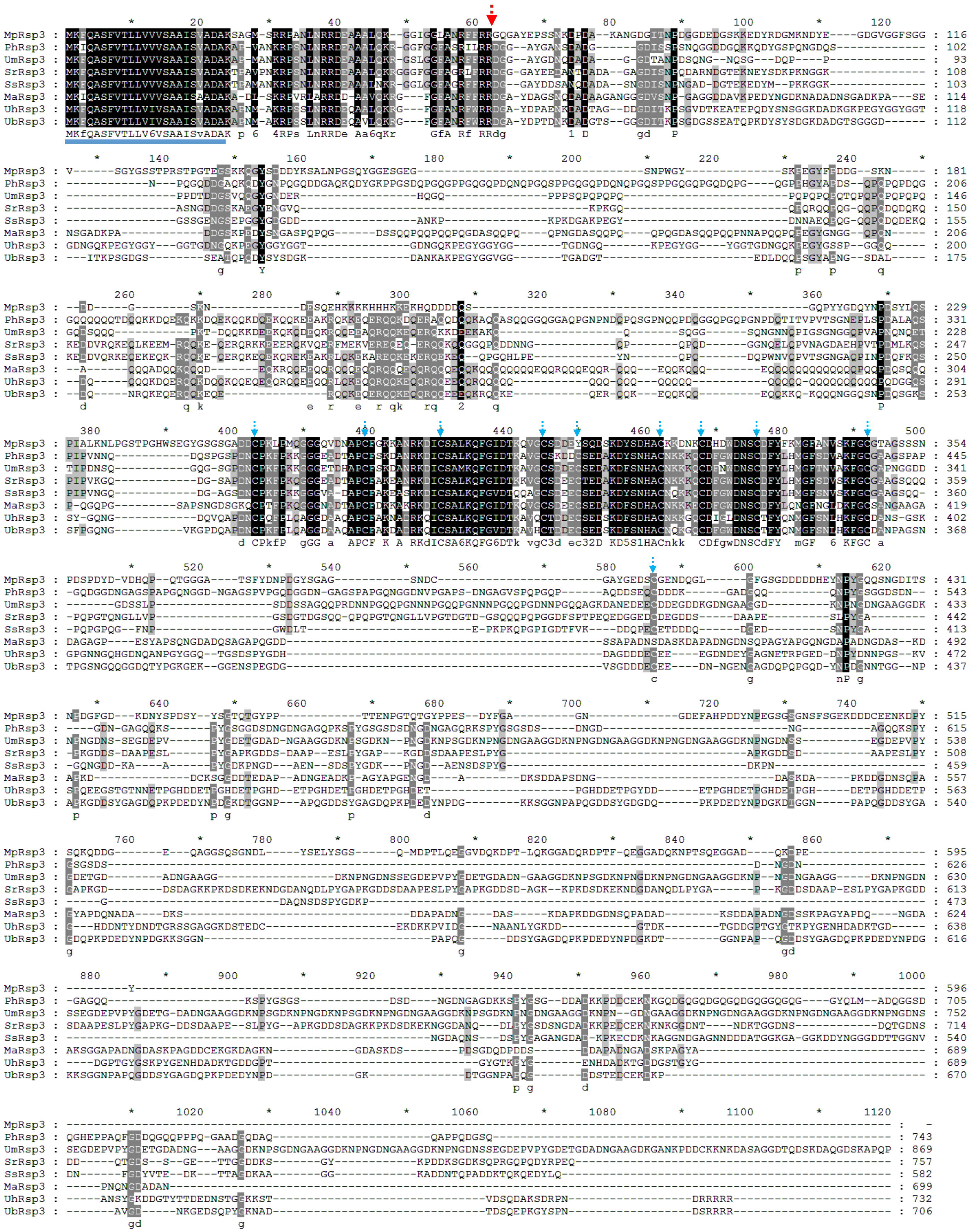

Supplementary Figure 3: Amino acid sequence alignment of Rsp3 orthologs from different smuts. Sequences of the full length proteins from *U. maydis* (UmRsp3), *Sporisorium reilianum* (SrRsp3), *Sporisorium scitamineum* (SsRsp3), *Pseudozyma hubeiensis* (PhRsp3), *Melanopsichium pennsylvanicum* (MpRsp3), *Ustilago hordei* (UhRsp3), *Ustilago bromivora* (UbRsp3), and *Moesziomyces antarcticus* (MaRsp3) were obtained from the public databases. Conserved amino acids are highlighted in black. The signal peptide of UmRsp3 is indicated by a blue line. The identified cleavage site in UmRsp3 is indicated by a red arrow. The conserved cysteines are indicated by blue arrows.

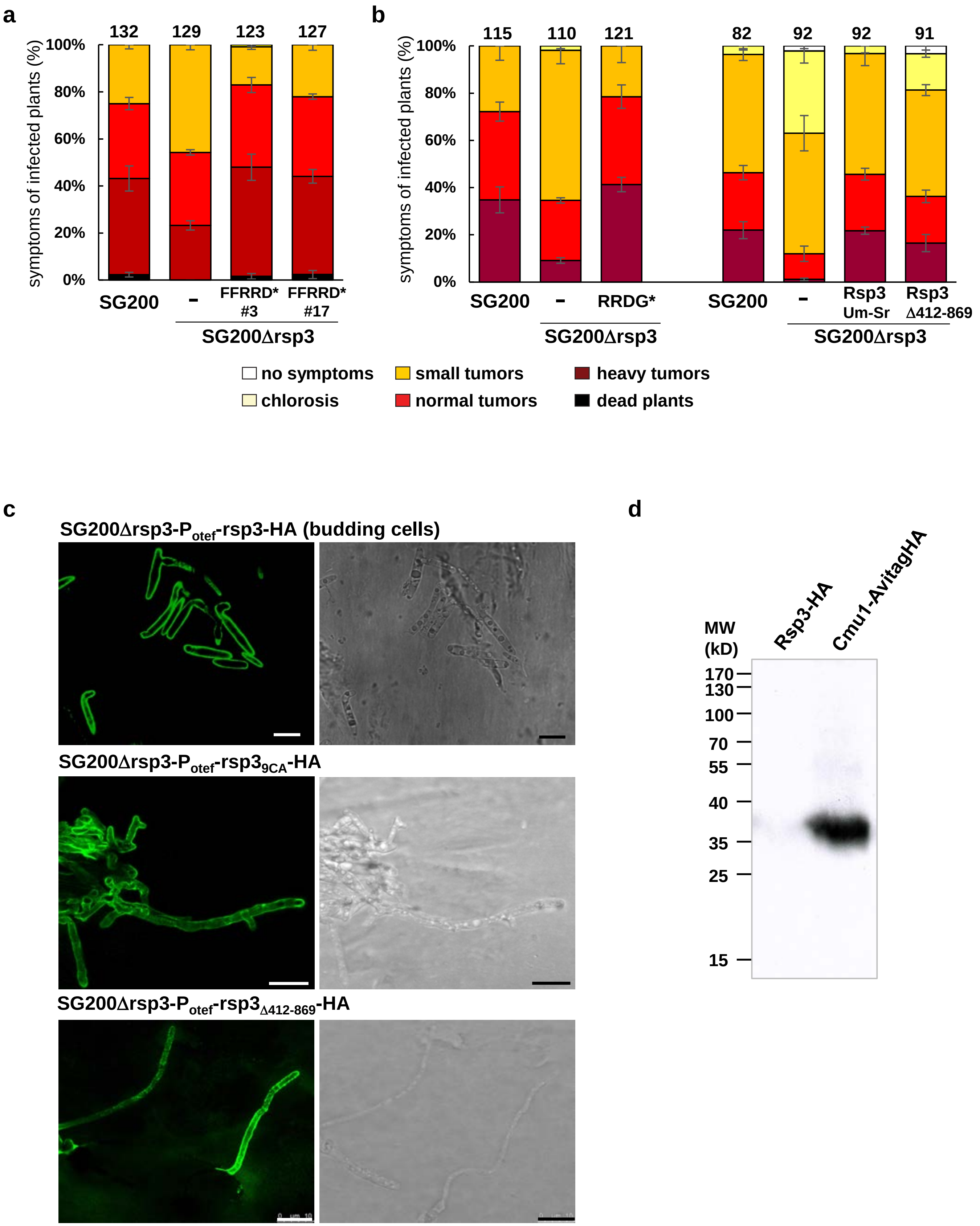
a
132
129
123
127
100%
80%
60%
40%
20%
0%
symptoms of infected plants (%)
SG200
-
FFRRD* #3
FFRRD* #17
SG200Δrsp3
b
115
110
121
82
92
92
91
100%
80%
60%
40%
20%
0%
symptoms of infected plants (%)
SG200
-
RRDG*
SG200Δrsp3
SG200
-
Rsp3 Um-Sr
Rsp3 Δ412-869
SG200Δrsp3
no symptoms
small tumors
heavy tumors
chlorosis
normal tumors
dead plants
c
SG200Δrsp3-Potef-rsp3-HA (budding cells)
SG200Δrsp3-Potef-rsp39CA-HA
SG200Δrsp3-Potef-rsp3Δ412-869-HA
d
MW (kD)
Rsp3-HA
Cmu1-AvitagHA
170
130
100
70
55
40
35
25
15

Supplementary Figure 4: Biological activity of Rsp3 proteins carrying mutations and binding to *U. maydis cells.* (**a, b**) Virulence of the indicated strains was assessed. Two independent strains were tested in (a). Disease symptoms were scored at 12 dpi following the disease rating depicted below. Three independent infections were done and data were combined. Standard deviation for each symptom category is shown. Total numbers of infected plants from the three biological replicates are indicated above the respective columns. (**c**) In the indicated strains Rsp3-HA and mutant proteins are expressed constitutively from the *otef* promoter. Strains were treated with hydroxy-fatty acids and sprayed on parafilm to induce filamentation. To visualize HA-tagged proteins, cells were immunostained with an anti-HA antibody and an AF488-conjugated secondary antibody without prior permeabilization. In the top panel a section was chosen where cells were budding, in the middle and lower panel filamentous cells were chosen. Bars: 10 µm. (**d**) Apoplastic fluid was collected at 3 dpi from maize leaves infected with SG200Δrsp3-rsp3-HA or SG200-cmu1-AvitagHA. Apoplastic fluid was TCA-precipitated and analyzed by western blotting using the anti-HA antibody. The experiment was repeated two times and one of these experiments is shown.

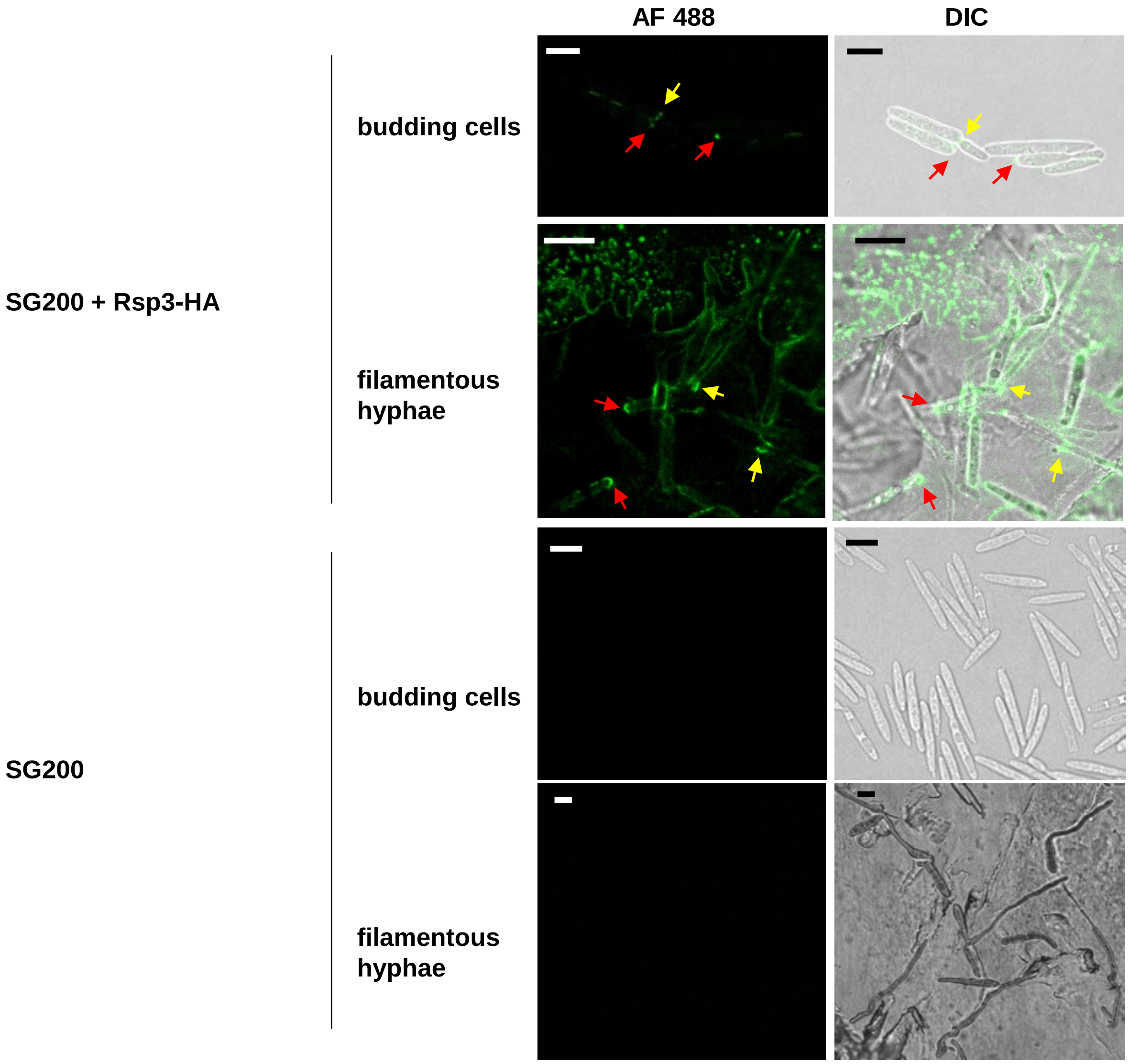

Supplementary Figure 5: Purified Rsp3-HA can attach to the surface of *U. maydis* cells. Rsp3-HA from the culture supernatant of SG200Δrsp3-P_{otef}-rsp3-HA was purified using HA-agarose beads and added either to budding cells of *U. maydis* SG200 (grown in liquid culture) or filamentous SG200 cells (treated with hydroxy-fatty acids and sprayed on parafilm). Budding as well as filamentous cells without Rsp3-HA added are shown as controls. To visualize Rsp3-HA, cells were immunostained with an anti-HA antibody and an AF488-conjugated secondary antibody without prior permeabilization. Red arrows indicate the hyphal tips and budding cell poles. Yellow arrows indicate septa. Bars: 10 µm.

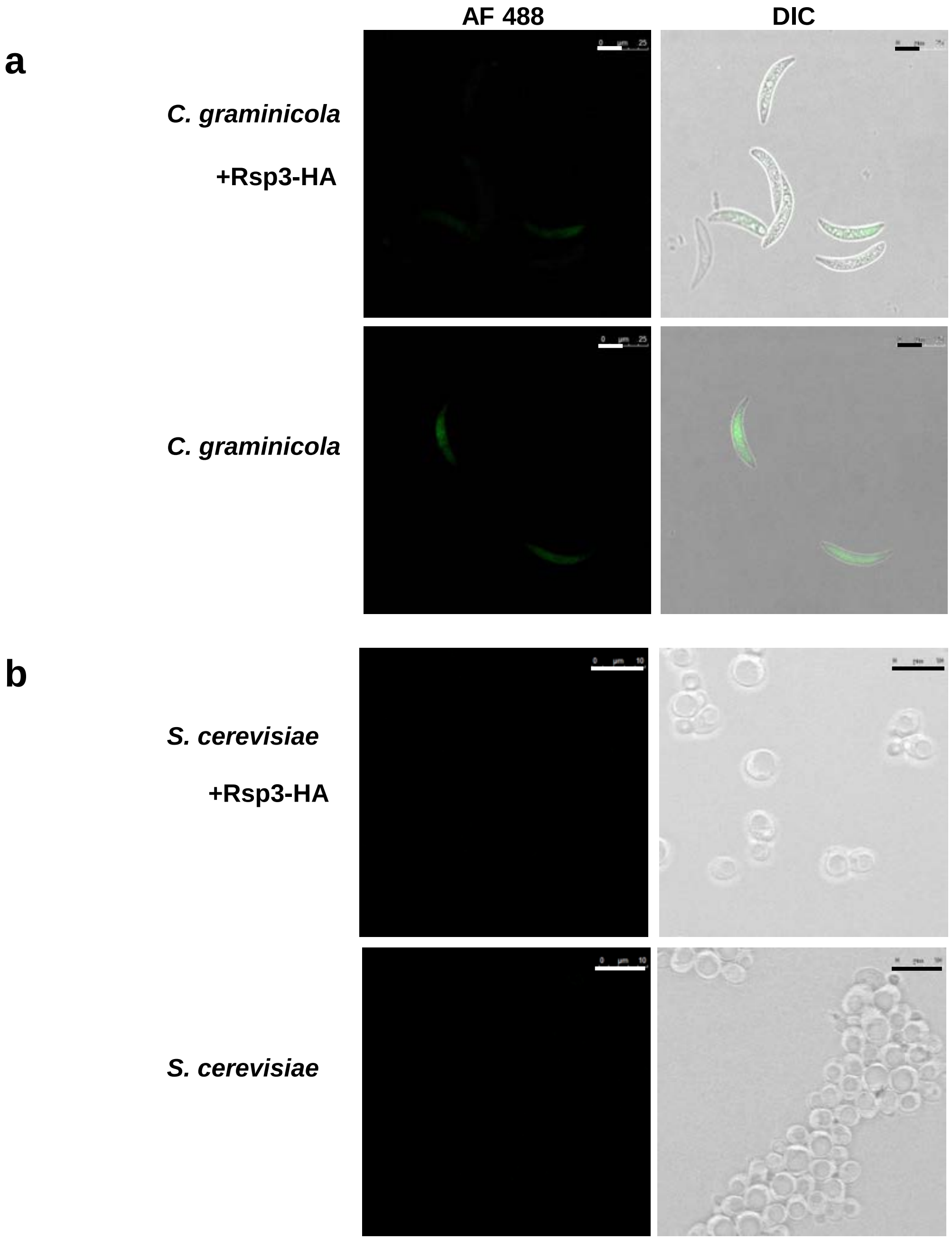

Supplementary Figure 6: Purified Rsp3-HA does not attach to conidia of *C. graminicola* or *S. cerevisiae*. Rsp3-HA from the culture supernatant of SG200Δrsp3-P_{otef}-rsp3-HA was purified using HA-agarose beads. (a) Rsp3-HA was added to conidia of *C. graminicola* M2 (CgM2). CgM2 cells without added Rsp3-HA are shown as control. To visualize Rsp3-HA, cells were immunostained with an anti-HA antibody and an AF488-conjugated secondary antibody without prior permeabilization. Bars: 10 µm. Some CgM2 cells show cytoplasmic autofluorescence of unknown origin, which is not associated with Rsp3-HA addition. (b) *S. cerevisiae* AH109 cells were incubated with or without Rsp3-HA addition followed by immunostaining of Rsp3-HA as in (a). Bars: 10 µm.

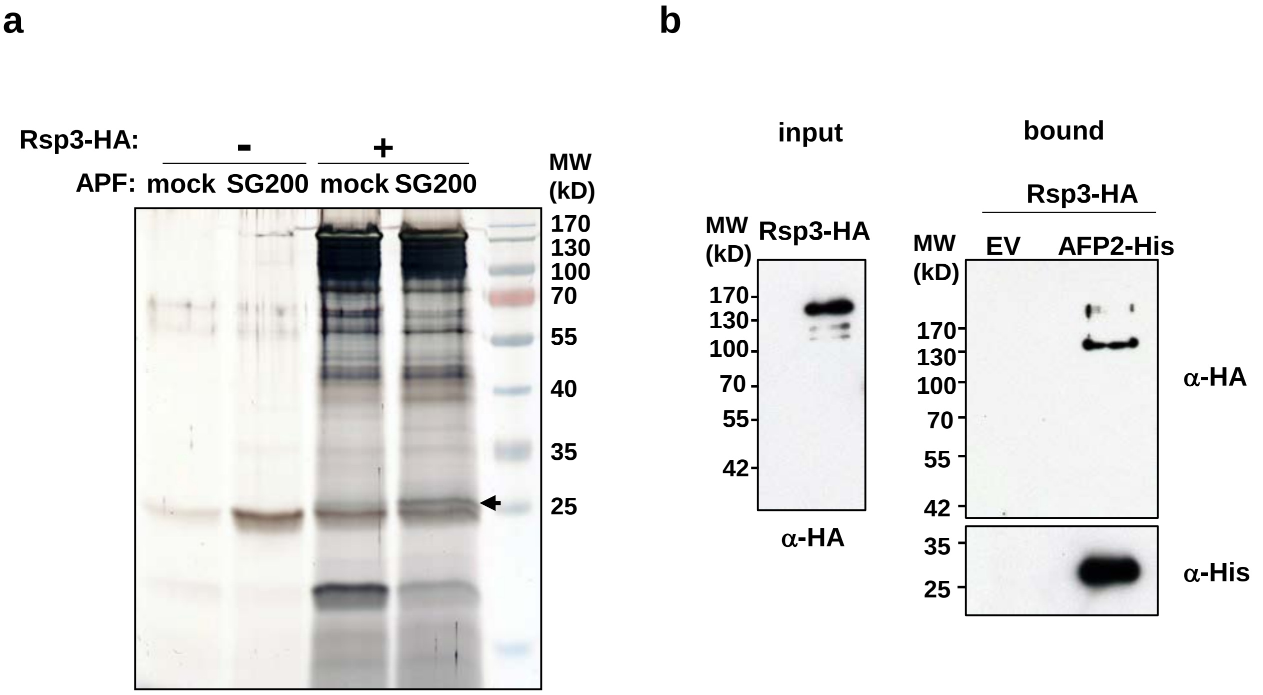

Supplementary Figure 7: Rsp3 interacts with maize secreted DUF26 domain containing proteins. (**a**) Apoplastic fluid collected from plant leaves inoculated with either SG200 or mock (H_2O) was incubated with or without Rsp3-HA purified from culture supernatants of SG200Δrsp3P_{otef}-rsp3-HA. After affinity purification on HA-agarose beads, proteins bound to the beads were eluted, separated by SDS-PAGE and silver-stained. The arrow indicates the band subjected to mass spectrometry analysis. (**b**) Rsp3 interacts with AFP2-His. *N. benthamiana* leaf tissue was infiltrated with *A. tumefaciens* carrying the AFP2-His expressing plasmid pICH-AFP2-His. As negative control infiltration was done with *A. tumefaciens* carrying the empty vector pICH31070 (EV). Leaves were homogenized and subjected to the same NTA-affinity purification steps. The beads bound by AFP2-His or EV, respectively, were mixed with Rsp3-HA from culture supernatant of SG200Δrsp3P_{otef}-rsp3-HA (input). Input and bound proteins were detected by western blot using anti-HA antibodies (top) and anti-His-HRP (bottom).

a

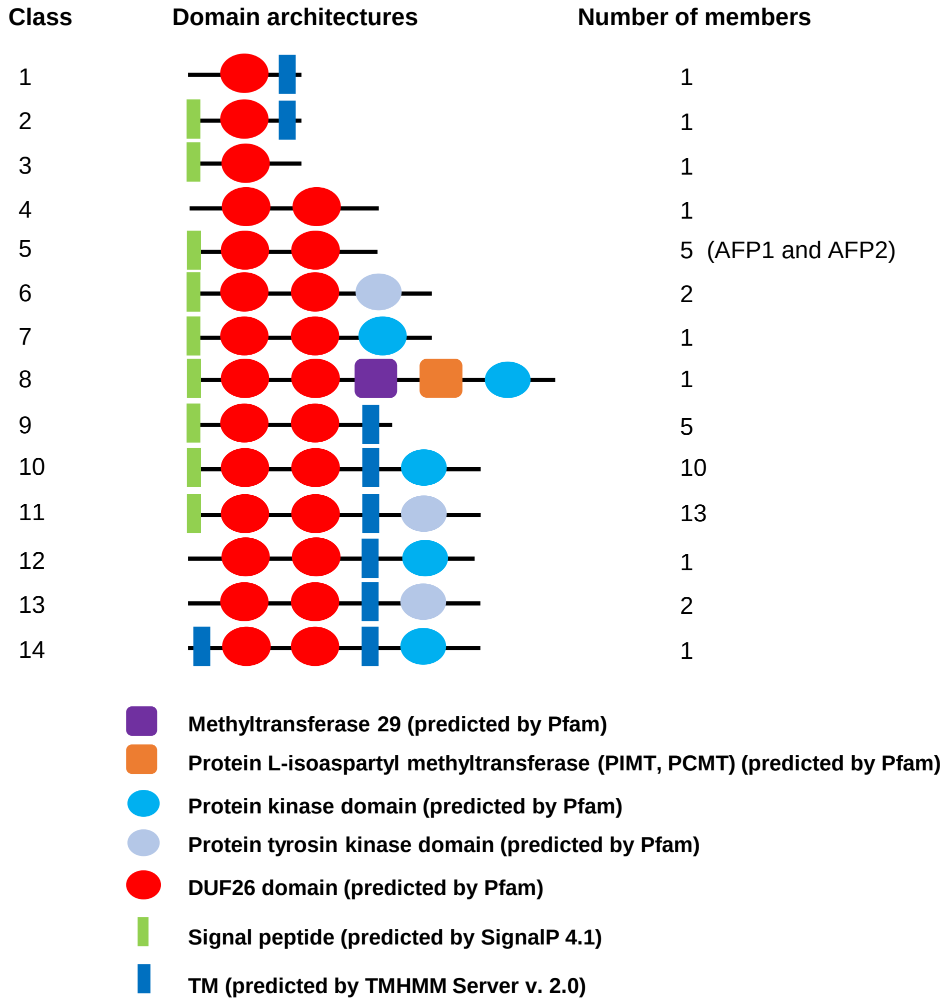
Class
Domain architectures
Number of members
1
2
3
4
5
6
7
8
9
10
11
12
13
14
1
1
1
1
5 (AFP1 and AFP2)
2
1
1
5
10
13
1
2
1
Methyltransferase 29 (predicted by Pfam)
Protein L-isoaspartyl methyltransferase (PIMT, PCMT) (predicted by Pfam)
Protein kinase domain (predicted by Pfam)
Protein tyrosin kinase domain (predicted by Pfam)
DUF26 domain (predicted by Pfam)
Signal peptide (predicted by SignalP 4.1)
TM (predicted by TMHMM Server v. 2.0)

b

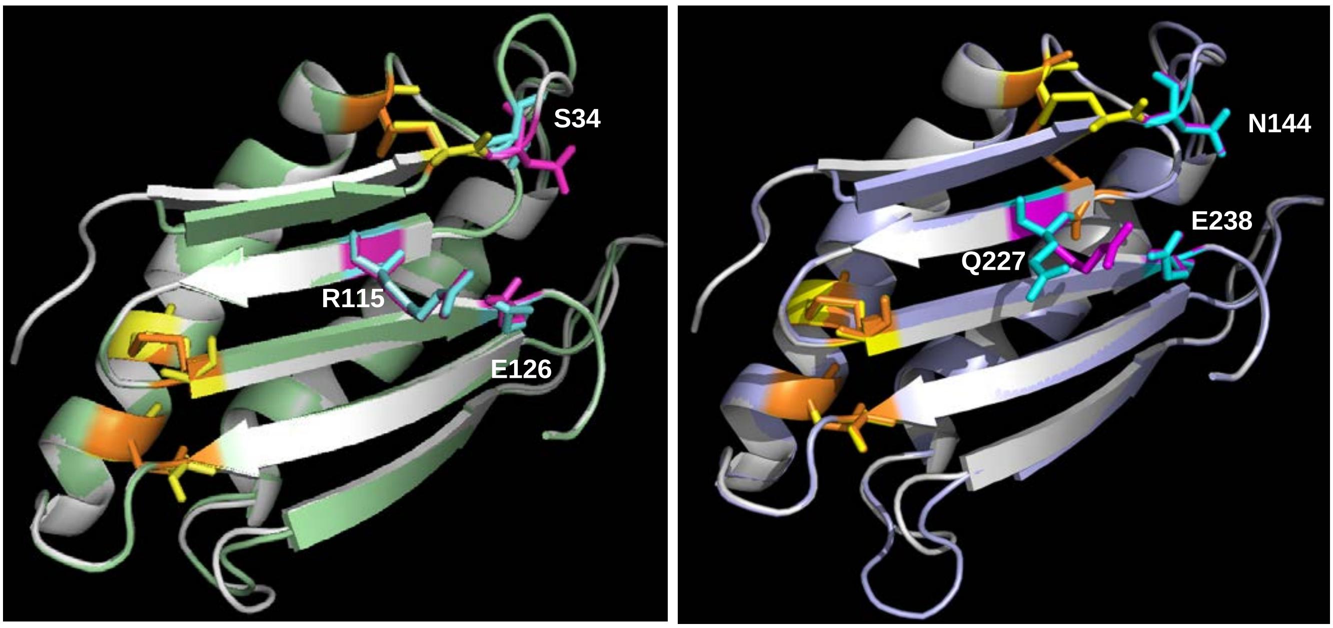
S34
R115
E126
N144
E238
Q227

c

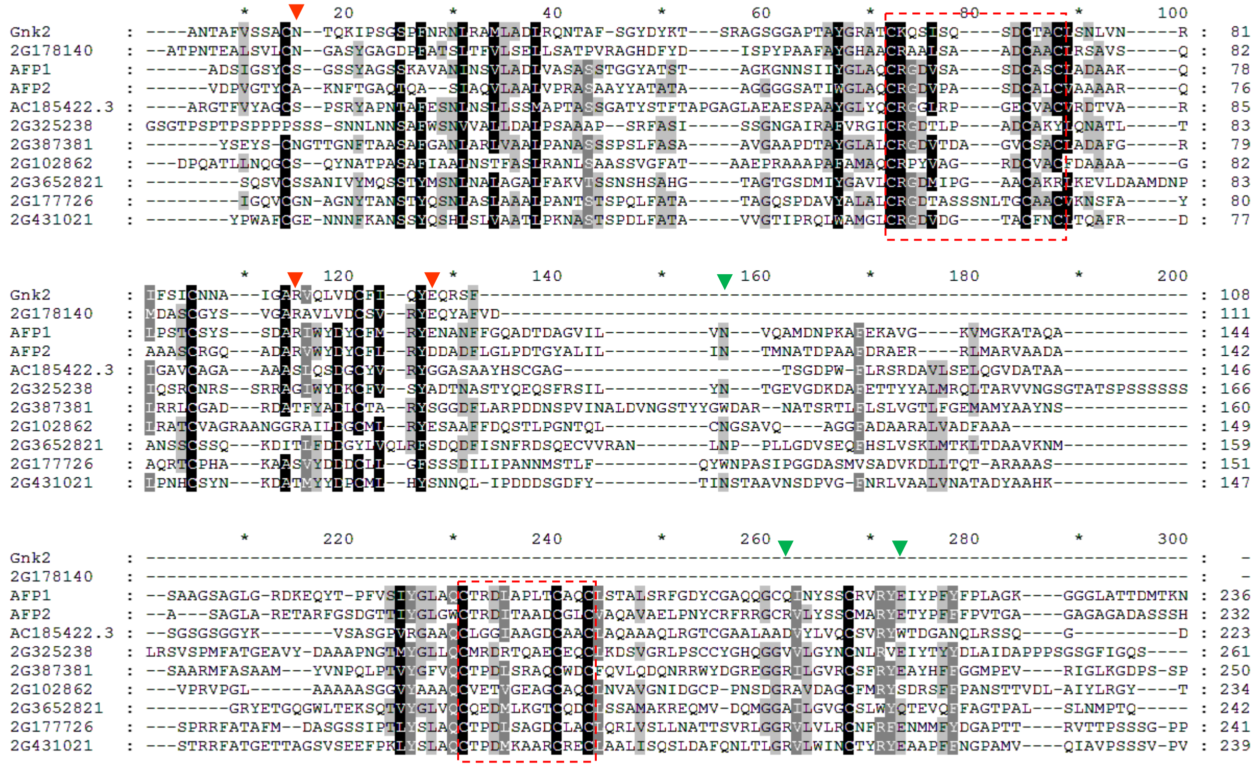

Supplementary Figure 8: DUF26-domain family proteins in maize. (**a**) Proteins containing the DUF26 domains (PF01657) in maize B73 were identified using Pfam predictions. They were grouped into fourteen classes according to their domain architecture, i.e number of predicted DUF26 domains, secretion signal, transmembrane domain, kinase domain, or presence of other domains. Domains are shown in different colors using the scheme depicted below. Domain descriptions can be found in the PF01657 database (http://pfam.xfam.org/family/PF01657). The number of protein members for each class is indicated. (**b**) Homology-based comparison of AFP1 with the structure of Gnk2 (PDB ID: 4XRE). The N-terminal (left plane; shown in green) and C-terminal domains (right plane; shown in purple) of AFP1 were modeled separately using Swiss-Model (https://swissmodel.expasy.org/interactive) and predicted structures were superimposed with Gnk2 (gray) using PyMOL. The cysteine bridges are indicated in yellow for Gnk2 and in orange for the modeled AFP1 domains. The three mannose binding residues of Gnk2 are shown in magenta. The predicted mannose binding residues of AFP1 are shown in cyan. (**c**) Amino acid sequence alignment of the 10 maize DUF26 domain containing proteins predicted to be secreted with Gnk2 from *G. biloba*. The red and green arrows indicate residues involved in mannose binding in Gnk2 and AFP1. The two DUF26 domains (C-X_8-C-X_2-C) are marked by a dashed line.

a

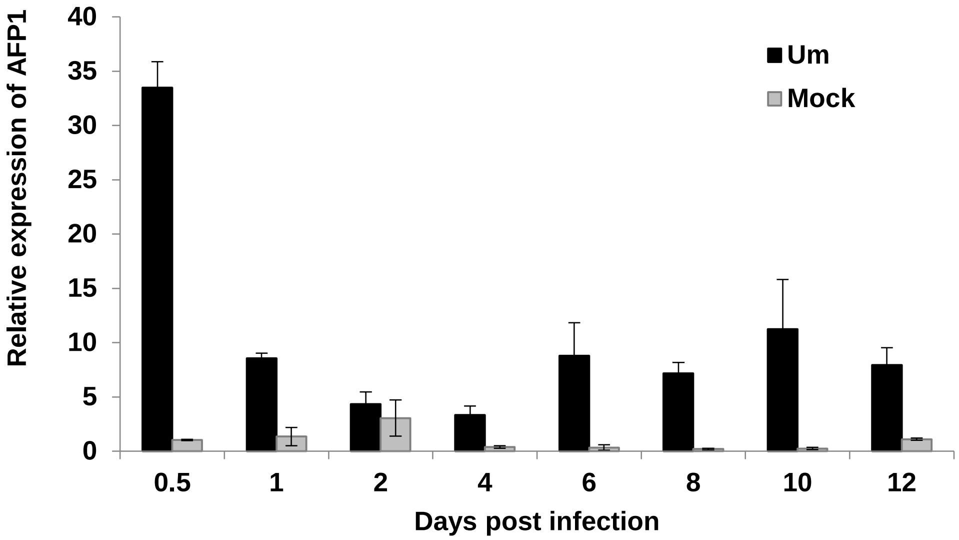
Relative expression of AFP1
40
35
30
25
20
15
10
5
0
Um
Mock
0.5
1
2
4
6
8
10
12
Days post infection

b

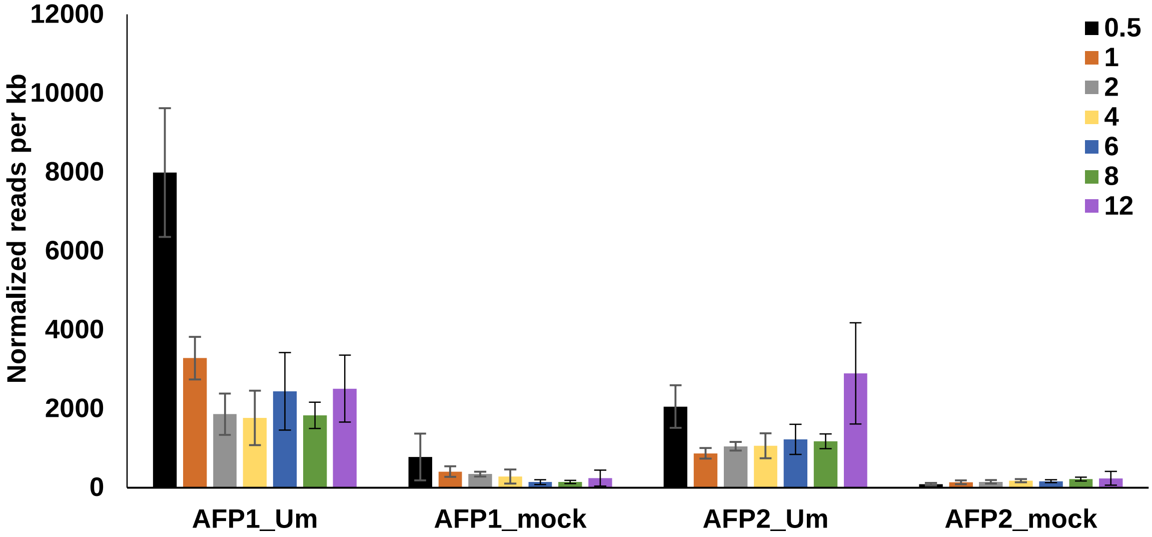
Normalized reads per kb
12000
10000
8000
6000
4000
2000
0
0.5
1
2
4
6
8
12
AFP1_Um
AFP1_mock
AFP2_Um
AFP2_mock

c1

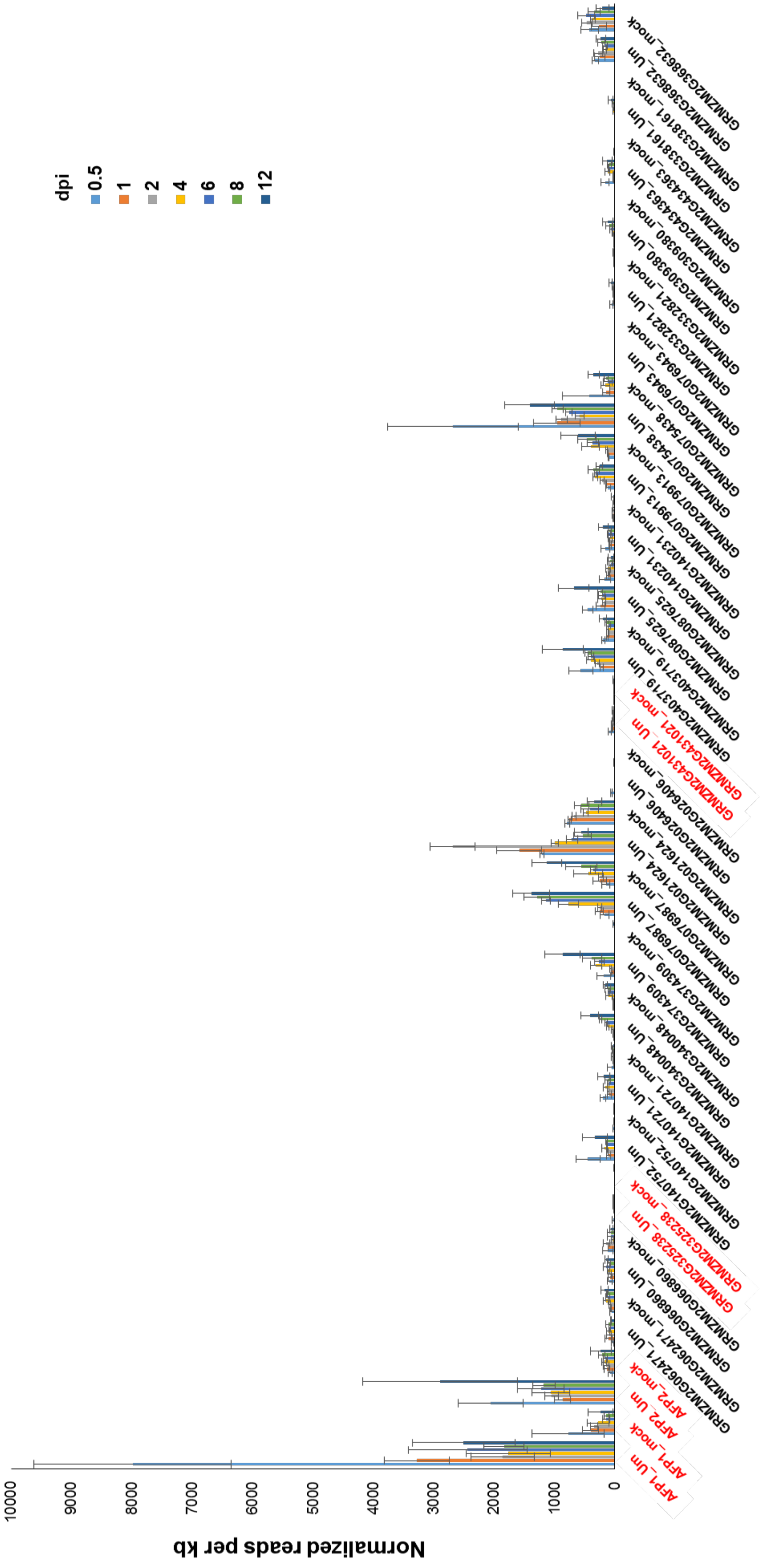
Normalized reads per kb
0
1000
2000
3000
4000
5000
6000
7000
8000
9000
10000
dpi
0.5
1
2
4
6
8
12
AFP1_Um
AFP1_mock
AFP2_Um
AFP2_mock
GRMZM2G062471_Um
GRMZM2G062471_mock
GRMZM2G066860_Um
GRMZM2G066860_mock
GRMZM2G325238_Um
GRMZM2G325238_mock
GRMZM2G140752_Um
GRMZM2G140752_mock
GRMZM2G140721_Um
GRMZM2G140721_mock
GRMZM2G340048_Um
GRMZM2G340048_mock
GRMZM2G374309_Um
GRMZM2G374309_mock
GRMZM2G076987_Um
GRMZM2G076987_mock
GRMZM2G021624_Um
GRMZM2G021624_mock
GRMZM2G026406_Um
GRMZM2G026406_mock
GRMZM2G431021_Um
GRMZM2G431021_mock
GRMZM2G403719_Um
GRMZM2G403719_mock
GRMZM2G087625_Um
GRMZM2G087625_mock
GRMZM2G140231_Um
GRMZM2G140231_mock
GRMZM2G079913_Um
GRMZM2G079913_mock
GRMZM2G075438_Um
GRMZM2G075438_mock
GRMZM2G076943_Um
GRMZM2G076943_mock
GRMZM2G332821_Um
GRMZM2G332821_mock
GRMZM2G309380_Um
GRMZM2G309380_mock
GRMZM2G434363_Um
GRMZM2G434363_mock
GRMZM2G338161_Um
GRMZM2G338161_mock
GRMZM2G368632_Um
GRMZM2G368632_mock

c2

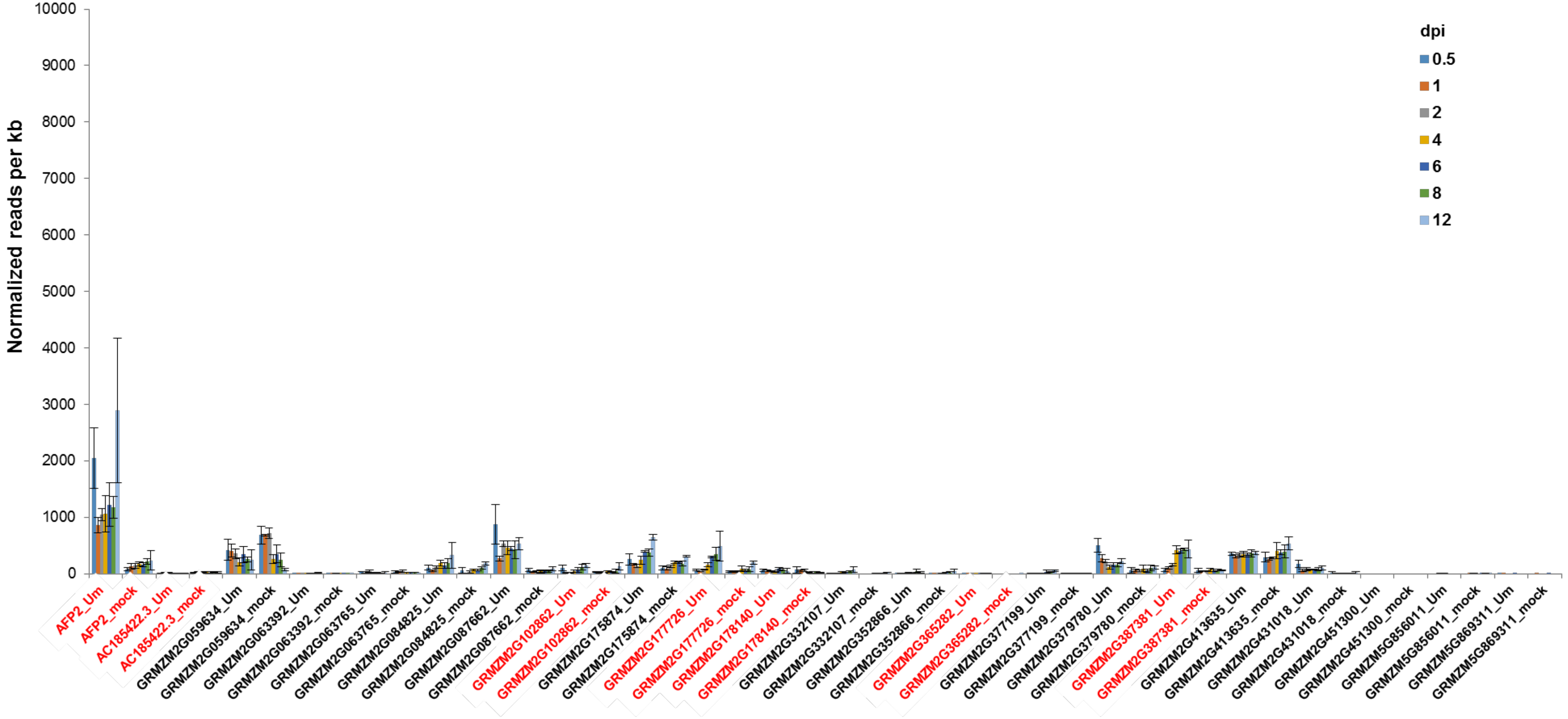

Normalized reads per kb
10000
9000
8000
7000
6000
5000
4000
3000
2000
1000
0
dpi
0.5
1
2
4
6
8
12
AFP2_Um
AFP2_mock
AC185422.3_Um
AC185422.3_mock
GRMZM2G059634_Um
GRMZM2G059634_mock
GRMZM2G063392_Um
GRMZM2G063392_mock
GRMZM2G063765_Um
GRMZM2G063765_mock
GRMZM2G084825_Um
GRMZM2G084825_mock
GRMZM2G087662_Um
GRMZM2G087662_mock
GRMZM2G102862_Um
GRMZM2G102862_mock
GRMZM2G175874_Um
GRMZM2G175874_mock
GRMZM2G177726_Um
GRMZM2G177726_mock
GRMZM2G178140_Um
GRMZM2G178140_mock
GRMZM2G332107_Um
GRMZM2G332107_mock
GRMZM2G352866_Um
GRMZM2G352866_mock
GRMZM2G365282_Um
GRMZM2G365282_mock
GRMZM2G377199_Um
GRMZM2G377199_mock
GRMZM2G379780_Um
GRMZM2G379780_mock
GRMZM2G387381_Um
GRMZM2G387381_mock
GRMZM2G413635_Um
GRMZM2G413635_mock
GRMZM2G431018_Um
GRMZM2G431018_mock
GRMZM2G451300_Um
GRMZM2G451300_mock
GRMZM5G856011_Um
GRMZM5G856011_mock
GRMZM5G869311_Um
GRMZM5G869311_mock

d

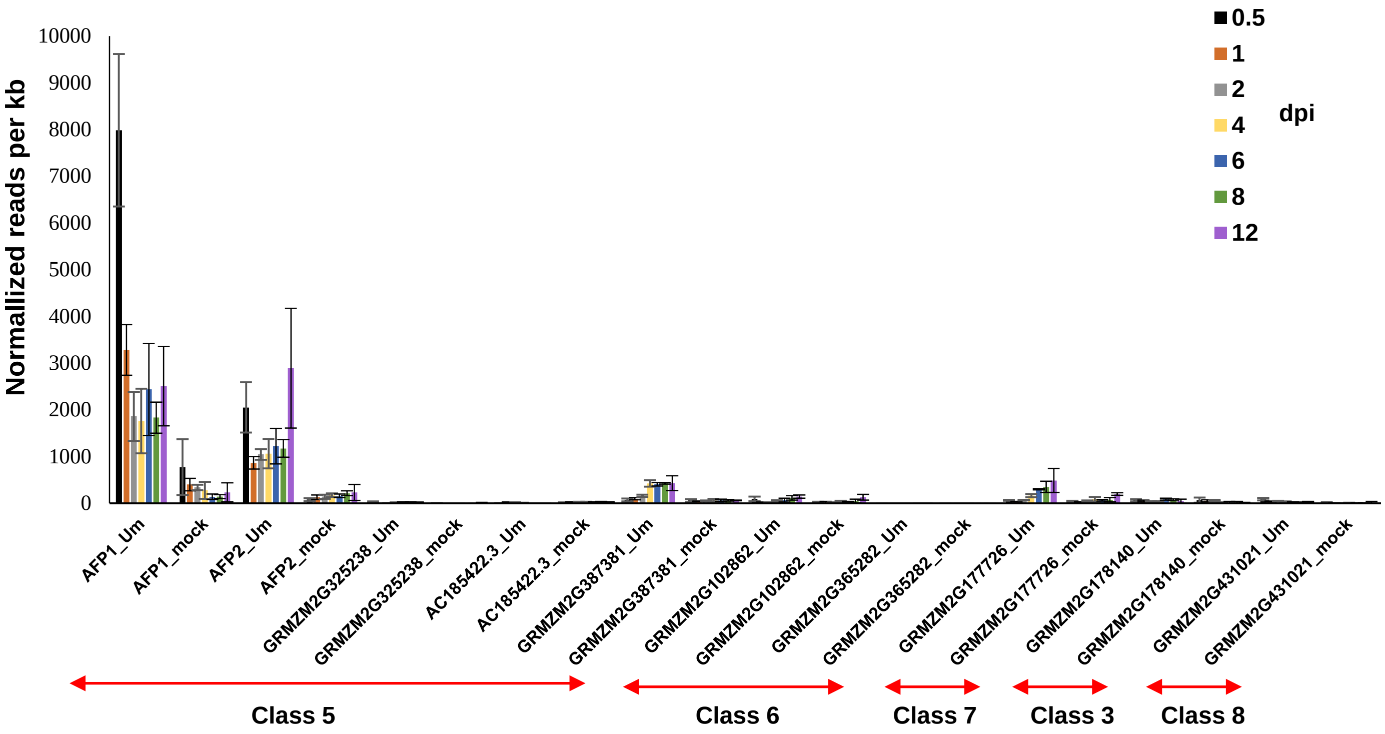

Supplementary Figure 9: Expression analysis of DUF26 domain containing proteins from maize. (**a**) qRT-PCR analysis of AFP1. RNA was prepared from Early Golden Bantam plants infected with a mixture of FB1 and FB2 (Um) or inoculated with water (Mock) as control. Infected material and corresponding material from the mock-inoculated control plants was collected between 0.5 and 12 days post infection (dpi). RNA was extracted and analyzed by qRT-PCR for APF1 expression. The expression of AFP1 in the 0.5 dpi mock sample was set to 1.0. The expression levels of AFP1 were normalized relative to the maize GAPDH gene. Three biological replicates were analyzed. Error bars indicate standard deviations. Values represent mean ± s.d from three replicates. The RNA samples have been used previously for an RNAseq analysis by Lanver et al. (2018)[1]. (**b**) RNAseq expression profiles of AFP1 and AFP2 after infection. Data were compiled from Lanver et al. (2018)[1]. (**c1 and c2**) RNAseq expression profiles of the 45 maize genes predicted to encode DUF26-domain proteins. Data were compiled from Lanver et al (2018)[1]. Maize genes predicted to encode secreted DUF26-domain containing proteins are indicated in red. (**d**) RNAseq expression profiles of all predicted secreted DUF26 domain proteins of maize. The affiliation to different protein classes depicted in Supplementary Figure 8a is indicated below. Data were compiled from Lanver et al. (2018)[1] .

a

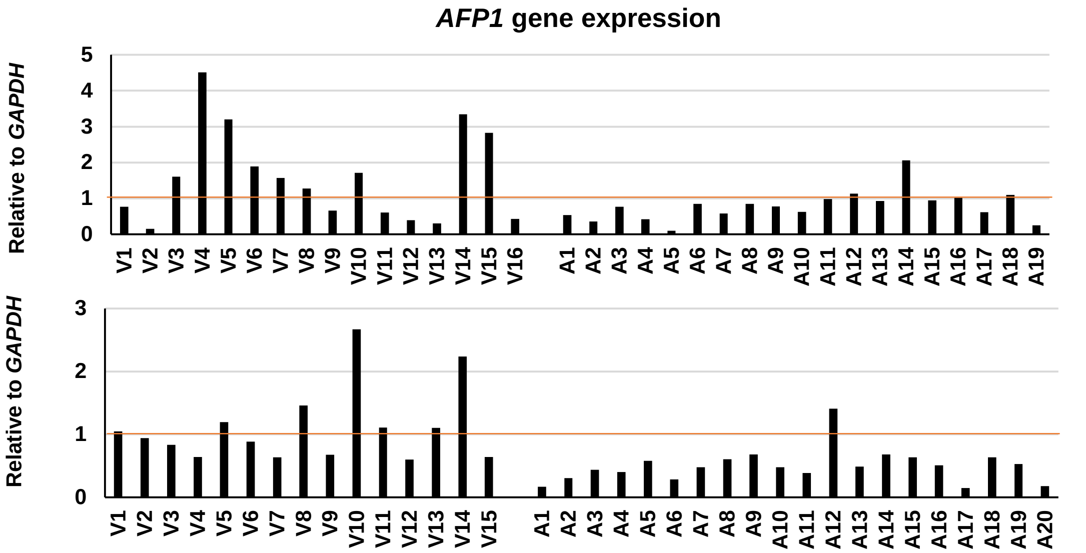

b

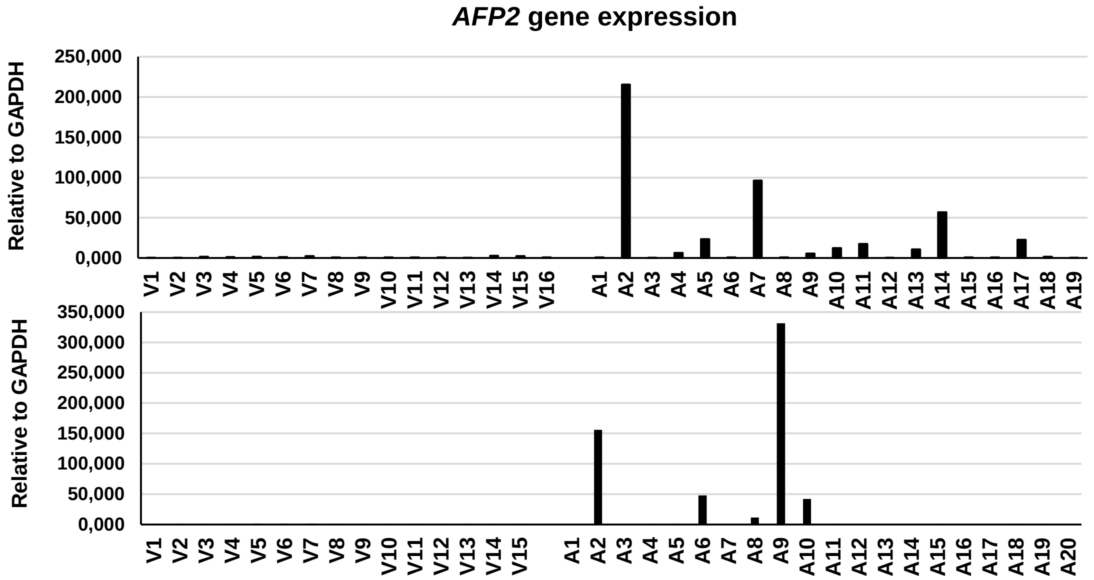

Supplementary Figure 10: Expression analysis of AFP1 and AFP2 in individual silenced plants. RNA was prepared from maize leaf samples that were inoculated either with FoMV viral sap expressing both AFP1 and AFP2 silencing constructs (A: FoMVsAFP1/2) or FoMV viral sap without silencing constructs as control (V: FoMV) and subsequently infected with SG200Δrsp3. Expression of AFP1 and AFP2 was determined by qRT-PCR. Expression levels of AFP1 or AFP2 were normalized relative to the constitutively expressed maize GAPDH gene. The average expression of AFP1 in plants inoculated with FoMV was set to 1.0 (brown line). Two biological replicates were performed. (**a**) Expression levels of AFP1 in silenced and no-silenced plants. The upper and lower panel show data from the two biological replicates. (**b**) Expression levels of AFP2 in silenced and no-silenced plants. The upper and lower panel show data from the two biological replicates.

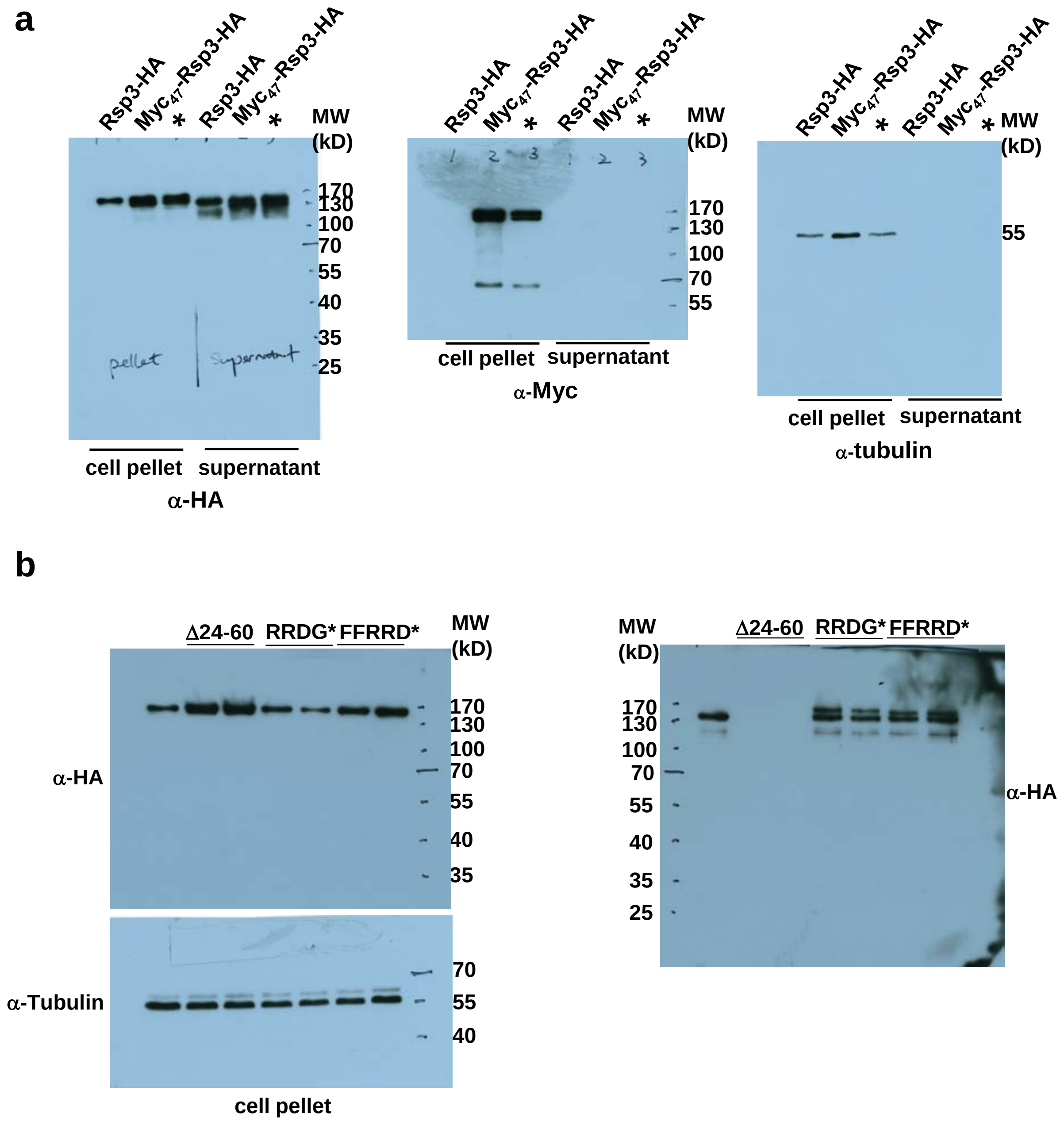

Supplementary Figure 11: Full scans of western blots from Figure 3. (a) Full scans of blots from Fig. 3b in the main text. (b) Full scans of blots from Fig. 3c in the main text. The asterisks indicate lanes with samples that were initially included in this study but that were deleted during the revision.

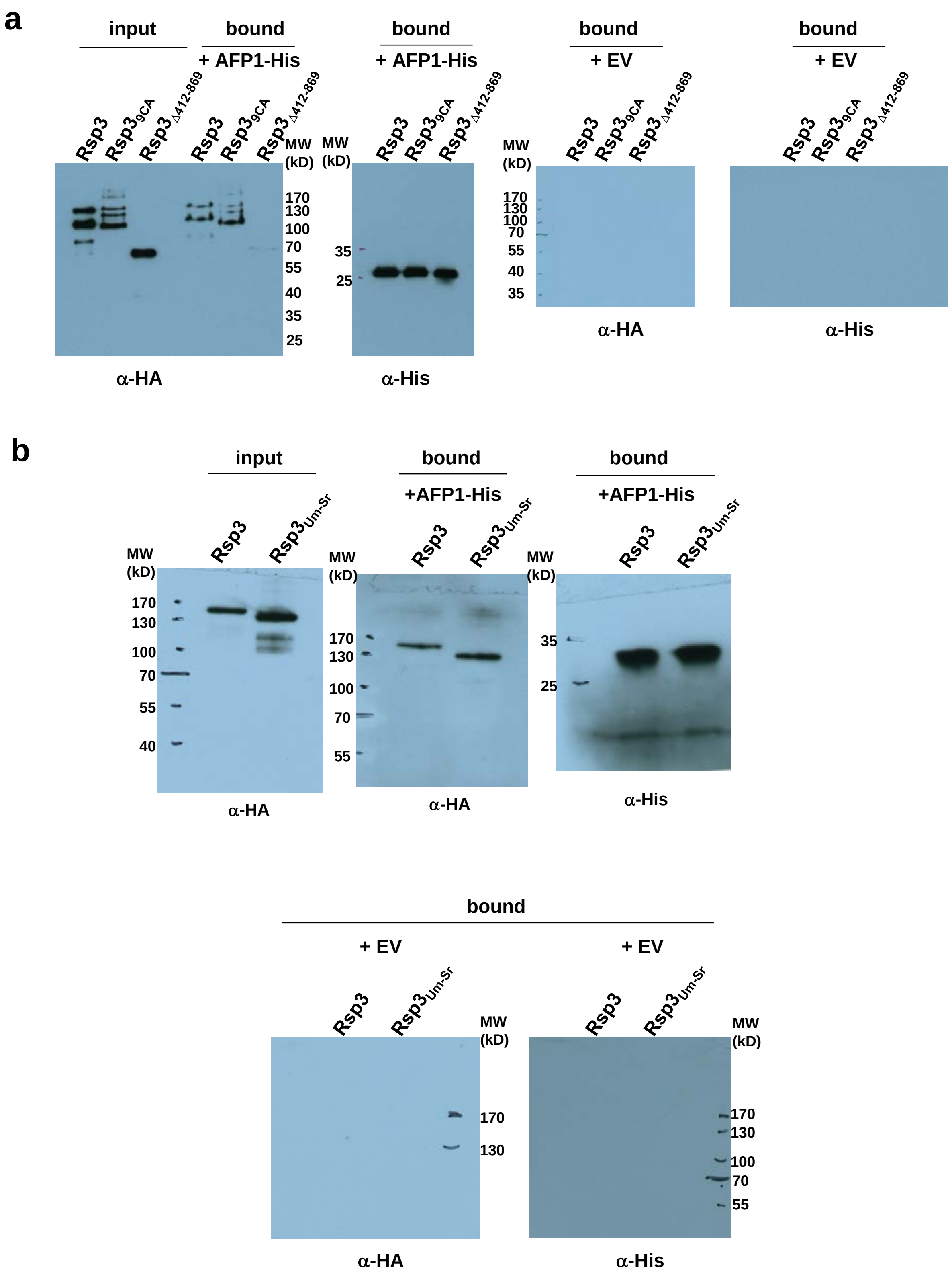

Supplementary Figure 12: Full scans of western blot from Figure 5. (a) Full scans of blots from Fig. 5a in the main text. (b) Full scans of blots from Fig. 5b in the main text.

a

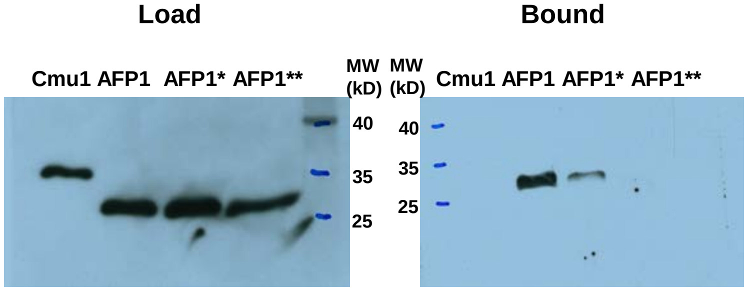

b

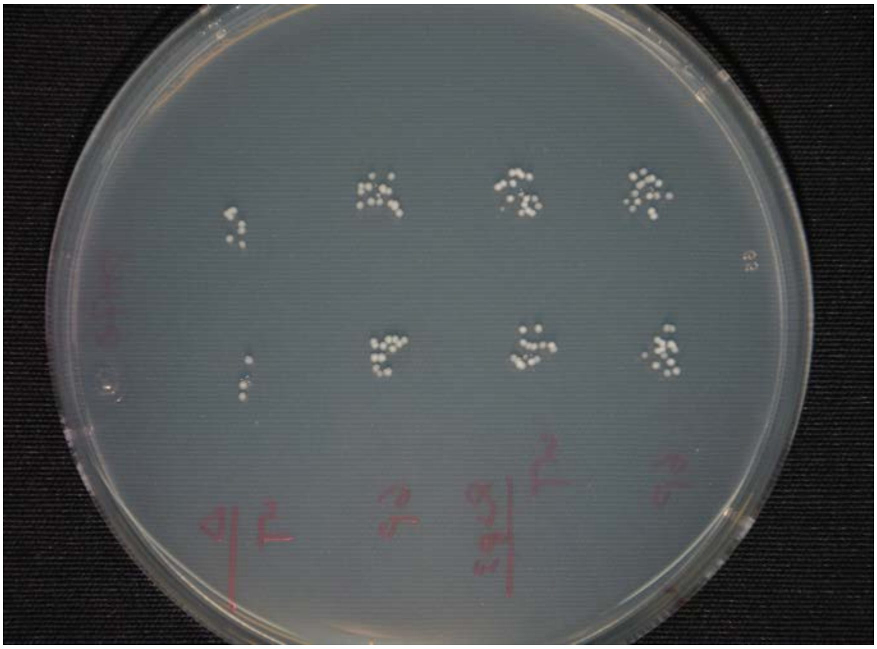

Supplementary Figure 13: Full scans of western blots and the full photograph from Figure 6. (a) Full scans of blots from Fig. 6b in the main text. (b) Full photograph from Fig. 6c in the main text.

Supplementary Table 1: Visualization of Rsp3-HA by immunogold labeling in infected maize tissue.

	**SG200[a]**	**SG200Δrsp3-rsp3HA[a]**
**Fungal Cytosol[b]**	0.074 ± 0.05	4.5 ± 1.3 ***[c]
**Biotrophic Interface[b]**	0 ± 0	39.8 ± 5.4 ***[c]
**Plant Cytosol[b]**	0.16 ± 0.08	0.14 ± 0.06 ns
**Plant Nucleus[b]**	0.9 ± 0.27	0.27 ± 0.1 ns
**Plant Chloroplast[b]**	0.30 ± 0.15	0.06 ± 0.02 ns

[a] Maize seedlings were infected by SG200 (control) and SG200Δrsp3-rsp3HA. Samples were collected at 4 dpi.

[b] Data represents the amount of gold particles bound to HA per μm^2 in different cell compartments. Data are means ± SE.

[c] Significant differences to the control were calculated with the Mann Whitney U-test. *** indicate significance at the 0.001 levels of confidence. ns, not significant different. N≥20 for each cell compartment.

Supplementary Table 2: Plasmids and primers used in this study

Plasmids	Primer	Sequences (5´-3´)
Generating rsp3 mutant:		
pCR2-rsp3KO	Rsp3#1	GACTAACTGCTCTTCAAGCAACG
	Rsp3#2	TGAGGCCTGAGTGGCCGAGAAGAAGAGTCGAAGTTGTGG
	Rsp3#3	ATGACGGCAGCAAAGGCAGTGATGAGCAGCAGTCTTACG
	Rsp3#4	GTCTCGAGATCAAAGGGTGTACC
Complementation:		
prsp3	Rsp3#5	CACCATATGTGATCACAGATGATCATCTCATTGC
	Rsp3#6	AATTGCGGCCGCCTAAGGCTGCGGGGCCTTGGAGTCGCCCTGAGC
potef-rsp3-HA	HA-F	GTCGTAGGGATAGCCGCCCGAAGGCTGCGGGGCCTTGGAGTC
	HA-R	GTCCCCGACTATGCCTAGGCGGCCGCCCGGCTGCAGATC
pHA-rsp3	Rsp3#7	ATAACCTCGTTTCCCCAGCA
	Rsp3#8	TTACTAGTCGAGGCGTAGTCGGGCACGTCGTAGGGGTAGGCGTCAG
	Rsp3#9	CCGACTACGCCTCGACTAGTAAGGCCCCCATGGCCAACAAG
	Rsp3#10	CGATTGGCGAAGCCACCGAGACTACCACGCTTCTG
prsp3(Δcys)	Rsp3#11	ATAACCTCGTTTCCCCAGCA
	Rsp3#12	CCGGGGCCGCCCGAGGCGTAGTCGGGCACGTCGTAGGGGTAGCCGCCCGAGG
	Rsp3#13	GATCCCTCGGGCGGCTACCCCTACGACGTGCCCGACTACGCCTCGGGCGGCC
	Rsp3#14	CCCTTGTCGTCACCCTCATC
prsp3 (T6)	Rsp3#15	AAGGATCCATGAAGTTCCAAGCCTCGTTCG
	Rsp3#16	AATTGCGGCCGCCTAAGGCTGCGGGGCCTTGGAGTCGCCCTGAGC
prsp3$_{9CA}$ (T6)	Rsp3#17	TCGGGTCCGGACAACGCCCCCAAGTTCCCTAAG
	Rsp3#18	ACTGATCATGCCCCAGCCTTCTCCAAGGACGCC
	Rsp3#19	AACCGCAAGGACATCGCCTCTGCTATGAAGCAG
	Rsp3#20	ACCAAGGCCGTTGGGGCCTCCGACGACGAGTGC
	Rsp3#21	GCCTCCGACGACGAGGCCAGCGAGGACGCCAAG
	Rsp3#22	TTTTCGAACCACGCTGCCAACAAGAAGAAGCAG
	Rsp3#23	AACAAGAAGAAGCAGGCCGACTTCAACTGGGAC
	Rsp3#24	AACTGGGACAACAGCGCCGACTTCTACCTTCAC
	Rsp3#25	GTCGCTAAGTTTGGCGCCGGTGCCCCTAATGGC
potef-rsp3$_{\Delta 24-60}$-HA	Rsp3#26	CACAGACAACATCATCCACGG
	Rsp3#27	GGCGTCAGCTACCGAGATG
	Rsp3#28	CCATCTCGGTAGCTGACGCC<br>GATGGTGGCGCTTATGGC
	Rsp3#29	TGCTGCTGCGAGTCCTG
potef-Myc$_{47}$-rsp3-HA	Rsp3#30	CACAGACAACATCATCCACGG
	Rsp3#31	TATCTAGAGAGATCCTCTTCAGAGATCAACTTCTGTTCCTTCTGGAGAGCGGCAGCCT
	Rsp3#32	TATCTAGACGTGGTAGTCTCGGTGGCTT
	Rsp3#33	CGGGATCCACCCTTGTCGTCACCCTCAT
potef-Myc$_{47}$-rsp3(RRDG*)-HA	Rsp3#30	CACAGACAACATCATCCACGG

	Rsp3#34<br>Rsp3#35<br>Rsp3#36	ATAAGCGCCAGCAGCAGCAGCGAAGAAGCGATTGGCG<br>TGCTGCTGCTGGCGCTTATGGCGACAA<br>CAGATGTCCTTGCGGTTGGCGTCCTTGGAG
potef-Myc$_{47}$rsp3(FFRRD*)-HA	Rsp3#30<br>Rsp3#37<br>Rsp3#38<br>Rsp3#36	CACAGACAACATCATCCACGG<br>AGCAGCAGCAGCGCGATTGGCGAAGCCA<br>CAATCGCGCTGCTGCTGCTGCTGGTGGCGCTTAT<br>CAGATGTCCTTGCGGTTGGCGTCCTTGGAG
potef-rsp3$_{\Delta 412\text{-}869}$-HA	Rsp3#30<br>Rsp3#39	CACAGACAACATCATCCACGG<br>TATGCGGCCGCTAGGCATAGTCGGGGACGTCGTAGGGATAACCCTTGTCGTCACCCTCAT
potef-rsp3$_{\text{Um-Sr}}$-HA	Rsp3#40<br>Rsp3#41<br>Rsp3#42<br>Rsp3#43	GAGCAGAAGCGTCAACAG<br>CCA TGT GAA GGT AGA AGT CGC AGC TGT TGT CCC AGT TGA AG<br>GCGACTTCTACCTTCACATGG<br>CGATCTGCAGCCGGGCGGCCGCCTAGGCATAGTCGGGGACGTCGTAGGGATAGCCGCCCGACTGCTCCGGCCTGTAGTCCTG
pSr-rsp3.HA	Rsp3#44<br>Rsp3#45<br>Rsp3#46<br>Rsp3#47	CTCTATCCATTCCACGTTCTTG<br>GGATATCTAGACTGCTCCGGCCTGTAGTCCTGGG<br>CCGGAGCAGTCTAGATATCCCTACGACGTCCCCGACTAT<br>GAGAGGCGGTTTGCGTAT
Protein expression in N. benthamiana:<br>pICH-AFP1-His	AFP1#1<br>AFP1#2<br>AFP1#3<br>AFP1#4<br>AFP1#5<br>AFP1#6	TGCAGGTATGGAGTCCAGCAGG<br>AGCTGAGACCTTAGTGGTGGTGGTGGTGGTGAGGACGCACGACGATCTTGGTGTTC<br>GGAACCCTGTGGTTGGCACATAC<br>TGCTGGACTCCATACCTGCAACAATAAG<br>CCACCACTAAGGTCTCAGCTTGAAGACT<br>GCTCACTAGTCGATCTAGTAAC
pICH-AFP1*-His	AFP1#7<br>AFP1#8<br>AFP1#9	TACTGCGCAGGAAGCAGCTAC<br>TTCCGACGCGGCGATATGGTACGACTAC<br>TTCATGCGGTACGCGAACGCCAACTTC
pICH-AFP1**-His	AFP1#10<br>AFP1#11<br>AFP1#12	ATCCTGGTGGCAGTGCAGGCCATGGAC<br>GGCTGCGCGATCAACTACAGCAG<br>TGCGCTACGCGATCTACCCCTTCTAC
pICH-AFP2-His	AFP2#1<br>AFP2#2<br>AFP2#3<br>AFP2#4	GTTCTTATTGTTGCAGGTATGGCACCTCCACCTTG<br>TAGTGGTGATGGTGGTGATG<br>GCCATACCTGCAACAATAAGAAC<br>CATCACCACCATCACCACTAAGGTCTCAGCTTGAAGAC
FoMV-induced gene silencing		
pFOMV-AFP1	antiAFP1-F<br>antiAFP1-R	GCTCTAGAGTACCGCATGAAGCAGTAGTCGTACC<br>TAACTCGAGGCCGCGGACTCCATCGGCAGCTACTG
pFoMV-AFP2	antiAFP2-F<br>antiAFP2-R	GCTCTAGAGTAGGTCTCGTAGCGAGCCATGCAG<br>GCCTCGAGGCGCTCATCCTCATCAACACCATGAAC
q-RT-PCR	ppi-F<br>ppi-R<br>GAPDH-F	ACATCGTCAAGGCTATCG<br>AAAGAACACCGGACTTGG<br>CTTCGGCATTGTTGAGGGTTTG

	GAPDH-R	TCCTTGGCTGAGGGTCCGTC
	AFP1-F	GGGCTGCCAGATCAACTACAG
	AFP1-R	GCACGACGATCTTGGTGTTC
	AFP2-F	GCGCTCATCCTCATCAACACCATGAAC
	AFP2-R	TAATCTCGAGGCAGTCGGCGGCGGTGAT
rsp3 alleles from field isolated	Rsp3#48	GACTAACTGCTCTTCAAGCAACG
	Rsp3#49	GTCTCGAGATCAAAGGGTGTACC

Supplementary Table 3: *U. maydis* strains used in this study

Strain	Genotype	Reference
SG200	*a1 mfa2 bE1 bW2*	Kamper et al., 2006[2]
Irapuato 5 (I5, *U. maydis* strain 5 isolated from Irapuato, Mexico)	*na*	Paredes-Lopez O.; unpublished
Oaxaca 3 (O3, *U. maydis* strain 3 isolated from Oaxaca, Mexico)	*na*	Paredes-Lopez O.; unpublished
Pachura 2 (P2, *U. maydis* strain 2 isolated from Pachura, Mexico)	*na*	Paredes-Lopez O.; unpublished
Sinaloa 3 (S3, *U. maydis* strain 3 isolated from Sinaloa, Mexico)	*na*	Paredes-Lopez O.; unpublished
Tuloca 6 (T6, *U. maydis* strain 6 isolated from Tuloca, Mexico)	*na*	Paredes-Lopez O.; unpublished
SG200AN1	a1:mfa2 bW2bE1 $P_{um01779}$:gfp	Lanver et al., 2014[3]
SG200AN1Δrsp3	a1 mfa2 bE1 bW2 $P_{um01779}$:gfp Δumag03274(rsp3)	This study
*SG200AN1Δrsp3-rsp3$_{\Delta cys}$	a1 mfa2 bE1 bW2 $P_{um01779}$:gfp Δrsp3 $ip^{r}[P_{rsp3}$:rsp3$_{\Delta cys}$]ip^{s}	This study
SG200Δrsp3	*a1 mfa2 bE1 bW2 Δumag03274(rsp3)*	This study
*SG200Δrsp3-rsp3	a1 mfa2 bE1 bW2 Δrsp3 $ip^{r}[P_{rsp3}$:rsp3]ip^{s}	This study
*SG200Δrsp3-rsp3-HA	a1 mfa2 bE1 bW2 Δrsp3 $ip^{r}[P_{rsp3}$:rsp3-HA]ip^{s}	This study
*SG200Δrsp3-HA-rsp3	a1 mfa2 bE1 bW2 Δrsp3 $ip^{r}[P_{rsp3}$:HA-rsp3]ip^{s}	This study
*SG200Δrsp3-rsp3$_{\Delta cys}$	a1 mfa2 bE1 bW2 Δrsp3 $ip^{r}[P_{rsp3}$:rsp3$_{\Delta cys}$]ip^{s}	This study
*SG200Δrsp3-rsp3(T6)	a1 mfa2 bE1 bW2 Δrsp3 $ip^{r}[P_{rsp3}$:rsp3(T6)]ip^{s}	This study
*SG200Δrsp3-rsp3(T6)$_{9CA}$	a1 mfa2 bE1 bW2 Δrsp3 $ip^{r}[P_{rsp3}$:rsp3(T6)$_{9CA}$]ip^{s}	This study
*SG200Δrsp3-rsp3$_{\Delta 412\text{-}869}$-HA	a1 mfa2 bE1 bW2 Δrsp3 $ip^{r}[P_{rsp3}$:rsp3$_{\Delta 412\text{-}869}$-HA]ip^{s}	This study
SG200Δrsp3-Myc$_{47}$-rsp3(RRDG)-HA	a1 mfa2 bE1 bW2 Δrsp3 $ip^{r}[P_{rsp3}$:Myc$_{47}$-rsp3(RRDG*)-HA]ip^{s}	This study
SG200Δrsp3-Myc$_{47}$-rsp3(FFRRD)-HA	a1 mfa2 bE1 bW2 Δrsp3 $ip^{r}[P_{rsp3}$:Myc$_{47}$-rsp3(FFRRD*)-HA]ip^{s}	This study
#SG200Δrsp3-Potef-rsp3-HA	a1 mfa2 bE1 bW2 Δrsp3 $ip^{r}[P_{otef}$:rsp3-HA]ip^{s}	This study
#SG200Δrsp3-Potef-rsp3Δ$_{24\text{-}60}$-HA	a1 mfa2 bE1 bW2 Δrsp3 $ip^{r}[P_{otef}$:rsp3Δ$_{24\text{-}60}$-HA]ip^{s}	This study
#SG200Δrsp3-Potef-Myc$_{47}$-rsp3-HA	a1 mfa2 bE1 bW2 Δrsp3 $ip^{r}[P_{otef}$:Myc$_{47}$-rsp3-HA]ip^{s}	This study
#SG200Δrsp3-Potef-Myc$_{47}$-rsp3(RRDG*)-HA	a1 mfa2 bE1 bW2 Δrsp3 $ip^{r}[P_{otef}$:Myc$_{47}$-rsp3(RRDG*)-HA]ip^{s}	This study
#SG200Δrsp3-Potef-Myc$_{47}$-rsp3(FFRRD*)-HA	a1 mfa2 bE1 bW2 Δrsp3 $ip^{r}[P_{otef}$:Myc$_{47}$-rsp3(RRDG*)-HA]ip^{s}	This study
#SG200Δrsp3-Potef-rsp3$_{9CA}$-HA	a1 mfa2 bE1 bW2 Δrsp3 $ip^{r}[P_{otef}$:rsp3$_{9CA}$-HA]ip^{s}	This study
#SG200Δrsp3-Potef-rsp3$_{\Delta 412\text{-}869}$-HA	a1 mfa2 bE1 bW2 Δrsp3 $ip^{r}[P_{otef}$:rsp3$_{\Delta 412\text{-}869}$-HA]ip^{s}	This study
SG200-Potef-cmu1-HA	a1 mfa2 bE1 bW2Δcmu1 $ip^{r}[P_{otef}$:cmu1-HA]ip^{s}	Djamei et al., 2011[4]
*SG200-mCherry-AvitagHA	a1 mfa2 bE1 bW2 $ip^{r}[P_{cmu1}$:mCherry-AvitagHA]ip^{s}	Lo Presti et al, unpublished
*SG200-cmu1-AvitagHA	a1 mfa2 bE1 bW2 $ip^{r}[P_{cmu1}$:cmu1-AvitagHA]ip^{s}	Lo Presti et al, unpublished
#SG200Δrsp3-Potef-rsp3$_{Um\text{-}Sr}$-HA	a1 mfa2 bE1 bW2 Δrsp3 $ip^{r}[P_{otef}$:rsp3 $_{Um\text{-}Sr}$-HA]ip^{s}	This study
*SG200Δrsp3-Srrsp3-HA	a1 mfa2 bE1 bW2 Δrsp3 $ip^{r}[P_{rsp3}$:Srrsp3-HA]ip^{s}	This study

* Plasmids expressing Rsp3 under its native promoter were integrated into the ip locus of *U. maydis* in single copy.
# Plasmids that constitutively expressing Rsp3 under control of *otef* promoter were integrated into ip locus in multiple copies. Correct integration into the ip locus was confirmed by southern blot analysis. *na*: not analyzed.

Supplementary References

1. Lanver, D. et al. The biotrophic development of *Ustilago maydis* studied by RNAseq analysis. *Plant Cell* **25**, doi: 10.1105/tpc.17.00764 (2018).

2. Kämper, J. et al. Insights from the genome of the biotrophic fungal plant pathogen *Ustilago maydis*. *Nature* **444**, 97-101 (2006).

3. Lanver, D. et al. Plant surface cues prime *Ustilago maydis* for biotrophic development. *PLoS Pathog.* **10**, e1004272 (2014).

4. Djamei, A. et al. Metabolic priming by a secreted fungal effector. *Nature* **478**, 395-398 (2011)
